# ﻿A new species of Southeast Asian dwarf tarantula in the genus *Phlogiellus* Pocock, 1897, from Lao PDR (Theraphosidae, Selenocosmiinae)

**DOI:** 10.3897/zookeys.1247.155398

**Published:** 2025-07-22

**Authors:** Patipan Sriranan, Chaowalit Songsangchote, Odeth Sihavong, Phoukhanh Sayavongsa, Keolamphanh Sidavong, Lilammone Satakoun, Khamla Inkhavilay, Narin Chomphuphuang, Ray Gabriel

**Affiliations:** 1 Department of Entomology and Plant Pathology, Faculty of Agriculture, Khon Kaen University, Khon Kaen, Thailand; 2 Spider Excellence Center of Thailand, Khon Kaen University, Khon Kaen 40002, Thailand; 3 Department of Biology, Faculty of Science, Champasack University, Champasack, Lao People's Democratic Republic; 4 Research Academic and Service Office, National University of Laos, Vientiane, Lao People's Democratic Republic; 5 Arachnology Research Association, London, UK

**Keywords:** Distribution, Mygalomorphae, Tarantula, Taxonomy, Theraphosidae, *Yamia*

## Abstract

A new species of Southeast Asian dwarf tarantula, *Phlogielluskhampheng* Sriranan, Songsangchote & Chomphuphuang, **sp. nov.**, is described from Pakse, Champasack Province, Lao PDR. The species is placed within the *Yamia* group of the genus *Phlogiellus*, which is characterized by the absence of maxillary lyra. *Phlogielluskhampheng* Sriranan, Songsangchote & Chomphuphuang, **sp. nov.** can be distinguished from other species within the *Yamia* group by the unique morphology of the female spermathecae and the male embolus. The habitat and natural history of *P.khampheng* Sriranan, Songsangchote & Chomphuphuang, **sp. nov.** are also discussed, with specimens found in mixed deciduous forests near Pakse, Lao PDR, inhabiting various microhabitats such as soil walls, under rocks, and within tree hollows. An updated comparison of scopula characteristics and labial cuspule counts across *Phlogiellus* species highlights the variability of these traits and their limitations as diagnostic features. Molecular phylogenetic analyses and species delimitation methods (ABGD and ASAP) further support the recognition of *P.khampheng* Sriranan, Songsangchote & Chomphuphuang, **sp. nov.** as a distinct species.

## ﻿Introduction

Tarantulas belong to the family Theraphosidae Thorell, 1869, a dominant group within the suborder Mygalomorphae comprising 172 genera and 1,132 species (World Spider Catalog 2025). Within Theraphosidae, the subfamily Selenocosmiinae Simon, 1889 is notable for several distinctive features including multiple rows of strikers on the retrolateral side of the chelicerae, maxillary lyra on the prolateral maxilla (although this feature is absent in some species of the genus *Phlogiellus*), posterior sternal sigillae positioned away from the sternal margins, and more than 160 labial cuspules (West et al. 2012; Nunn et al. 2016). The Southeast Asian dwarf tarantula genus *Phlogiellus* Pocock, 1897, comprises small-sized tarantulas, with 28 species reported from various locations, including Indonesia, the Philippines, China, Hong Kong, Singapore, Myanmar, Malaysia, Borneo, Taiwan, Thailand, Vietnam, Cambodia, the Solomon Islands, and Papua New Guinea (specifically New Britain) (Nunn et al. 2016; World Spider Catalog 2025). West et al. (2012) conducted a cladistic analysis of morphological characteristics in the subfamily Selenocosmiinae, identifying several unique traits for the genus *Phlogiellus*, including 200–350 labial cuspules, posterior lateral spinnerets nearly as long as metatarsus IV, and a deep foveal groove though this was only apparently based on four named species which and may not fully reflect on the other species in the genus. Additionally, they synonymized the Southeast Asian dwarf tarantula genus *Yamia*Kishida 1920 with *Phlogiellus* Pocock, 1897. Building on this work, Nunn et al. (2016) revised the genus *Phlogiellus* and further refined its diagnostic features. These include the presence of a third claw on the tarsus of leg IV, 160–320 labial cuspules (except in *P.pelidnus*), a retrolateral keel on the male embolus, and a very deep, procurved foveal groove that is narrower than the width of the ocular tubercle (except in *P.orophilus*). Chomphuphuang et al. 2017; described *Phlogielluslongipalpus* from Thailand and disagreed with some of the comments in Nunn et al. (2016) (for full discussion see Chomphuphuang et al. (2017). A few years later Sivayyapram et al. (2020) described two new species namely *Phlogiellusdaweiensis* Sivayyapram & Warrit, 2020 from Myanmar and *Phlogiellusraveni* Sivayyapram &Warrit, 2020 from the Philippines. Sivayyapram et al. (2020) also discussed the features used in Nunn et al. (2016) (for full discussion see Sivayyapram et al. 2020). Most recently Bariev et al. 2024 described the new species *Phlogiellusbirulai* Bariev & Logunov, (2024) from Vietnam and mentioned earlier problems with the work of Nunn et al. (2016), giving a detailed account regarding the confusion and uncertain nomenclature with *Phlogiellussubinermis*. In this study, we describe a new species of Southeast Asian dwarf tarantula from Pakse, Champasack Province, Lao PDR with its natural history, and discuss the diagnostic characters of this genus.

## ﻿Materials and methods

### ﻿Morphological study

**Figure 1.**
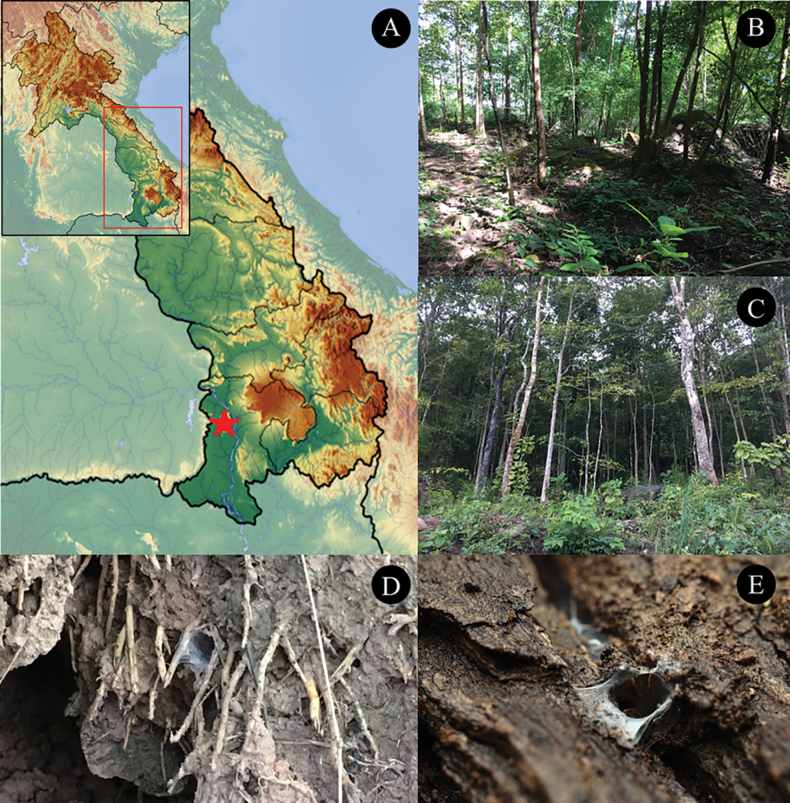
Locality of *Phlogielluskhampheng* sp. nov. **A.** Type locality; **B, C.** Habitat in the type locality, Pakse, Champasack Province, elevation 265 m; **D.** Habitat under a bamboo root cavity retreat. **E.** Habitat on timber.

All specimens were collected in Pakse, Lao PDR (Fig. 1) , and preserved in 95% ethanol. They were classified based on the methods of West et al. (2012), Nunn et al. (2016), Chomphuphuang et al. (2017) and Sivayyapram et al. (2020) by examining their morphology with a Nikon SMZ 745T stereomicroscope with measurements taken with digital vernier calipers. Diagnostic features were photographed using both a Nikon SMZ 745T and a Nikon SMZ25 stereomicroscope. Leg width and length measurements were taken from the right hand side of the specimen, following the methodology of Hamilton et al. (2016). Measurements of the male palp bulb and embolus were taken according to the guidelines set by Chomphuphuang et al. (2023). The number of claws and the division of the scopula in *Phlogiellus* species belonging to the *Yamia* group were compiled for comparison. Additionally, the number of labial cuspules and the body sizes of all described *Phlogiellus* species were also compiled for comparison. For comparison between the angle of the male palp specimens from Thailand and Lao PDR., see Chomphuphuang et al. (2023). All measurements are in millimeters.

All type and voucher specimens are deposited at the Department of Biology, Faculty of Natural Sciences, National University of Laos (NUOL), under the Streptaxis LA Unidentata collection, with the ID: NUoL00058–PKP0001–6. The following abbreviations are used in the text:

Eyes **AER** = Anterior eye row; **ALE** = Anterior lateral eyes; **AME** = Anterior median eyes; **MOA** = Median ocular area; **PER** = Posterior eye row; **PLE** = Posterior lateral eyes; **PME** = Posterior median eyes;

Spinnerets **PLS** = Posterior lateral spinnerets; **PMS** = Posterior median spinnerets;

Legs **Fem** = Femur; **Pat** = Patella; **Tib** = Tibia; **Met** = Metatarsus; **Tar** = Tarsus;

Bulb **ALH** = Angle between the lowest and highest point of the embolus; **ELC** = Embolus length along the curve; **ELS** = Embolus length along a straight line with the bulb; **EW** = Embolus width; PBL = Palp bulb length; **PBW** = Palp bulb width;

Institutes **NHMUK** = Natural History Museum, London, England; **MNHN** = Muséum National d’Histoire Naturelle, Paris, France; **MSNG** = Museo Civico di Storia Naturale ‘Giacomo Doria’, Genoa, Italy; **NHMW** = Naturhistorische, Wien, Austria.

### ﻿Molecular techniques and phylogenetic analyses

Genomic DNA was extracted from the coxa of the leg using either the Qiagen DNeasy Tissue Kit or the NucleoSpin Tissue Kit, following the respective protocols, and subsequently stored at -20 °C. For PCR amplification, a reaction mixture of 50 μl was prepared, comprising 20 μl of ultrapure water, 3 μl of DNA template, 1 μl of each primer (10 μM), and 25 μl of master mix. The thermal cycling protocol included an initial denaturation at 94 °C for 1 minute, followed by 40 cycles of denaturation at 94 °C for 30 seconds, annealing at 48 °C and 50 °C for 45 seconds, and extension at 72 °C for 1 minute. A final extension step was performed at 72 °C for 5 minutes. The COI gene fragment was amplified using the primers C1-J-1751 (forward: GAGCTCCTGATATAGCTTTTCC) and C1-N-2776 (reverse: GGATAATCAGAATATCGTCGAGG) as described by Hedin and Maddison (2001). The resulting PCR products were sent to U2Bio DNA sequencing services for sequencing using either Sanger or FastNGS methods.

The COI sequences underwent manual curation to ensure accuracy by verifying appropriate peak calls and contig assemblies, resulting in a polished dataset. Sequence alignment for each gene was carried out using the ClustalW algorithm (Thompson et al. 1994) with default settings in the MEGA 11 software (Tamura et al. 2021). Phylogenetic analysis was performed using the maximum likelihood (ML) method in IQ-TREE 2.2.0 (Minh et al. 2020), accessed via the IQ-TREE web server (Trifinopoulos et al. 2016). ModelFinder (Kalyaanamoorthy et al. 2017) within IQ-TREE was used to automatically determine the most appropriate substitution model for the dataset, incorporating the FreeRate heterogeneity model (+R). Clade support was evaluated using the SH-aLRT branch test (Guindon et al. 2010) and ultrafast bootstrap (UFBoot) analysis (Hoang et al. 2018).

### ﻿Species delimitation

Molecular data was used to delimit species through two distance-based approaches: Automatic Barcode Gap Discovery (ABGD) (Puillandre et al. 2012) and Assemble Species by Automatic Partitioning (ASAP) (Puillandre et al. 2021). Both analyses were performed using their respective web-based platforms, applying default settings and the Kimura 2-parameter (K2P) model (Kimura 1980).

### ﻿Species concept

The species concept we employ follows the Unified Species Concept, proposed by Kevin de Queiroz, is a framework that defines species as independently evolving metapopulation lineages. This concept synthesizes various traditional species concepts (e.g., Biological, Phylogenetic, and Morphological Species Concepts) under a single overarching framework by focusing on the shared fundamental criterion of evolutionary independence (De Queiroz 2007).

The study was based on two primary sources of data: (1) comparative materials directly examined from fresh specimens and (2) taxonomic references, which included photographs and morphological descriptions from relevant publications.

Comparative material examined:

*Phlogielluslongipalpus*Chomphuphuang et al. 2017: 1♂ (Holotype CUMZ–C2–NA1) Kamphaeng Phet province, Thailand, 1♀ (Paratype CUMZ-C4-NA4), 1♂ (Non-type B1–NA1: ENTOKKU). Lamphun province, Thailand and 1♀ (Non-type C8–CH2: ENTOKKU) Nakhon Nayok province, Thailand.

*Phlogiellusmoniqueverdezae*Nunn et al. 2016: 2♂ (Non-type P_mq001: ENTOKKU) Phang–Nga province, Thailand, (Non-type C6–VA1: ENTOKKU) Chumphon province, Thailand and 2♀ (Non-type T3–NA4: ENTOKKU). Koh Phayam, Ranong province, Thailand, (Non-type T3–NA5: ENTOKKU) Koh Lanta, Krabi province, Thailand.

*Phlogiellusaper* (Simon, 1891); 1♂ (Lectotype AR4675). Batavia, Java, MNHN.

*Phlogiellusbaeri* (Simon, 1877); 1♀ (Holotype AR 4671). Manila, Philippines, MNHN.

*Phlogiellusinsularis* (Simon, 1877): Juvenile (Holotype AR4579). Malamoy, Philippines, MNHN.

*Phlogiellusinermis* (Ausserer, 1871): 1♂, 1♀ (Lectotype AR4673). Java, MNHN.

The holotype, paratype, and other museum material examined are listed in the appendix within the Suppl. material 1; museum numbers are included where known.

Taxonomic authorities:

*Phlogiellusatriceps* Pocock, 1897c: 596, pl. 25, fig. 1.

*Phlogiellusatriceps* Nunn, West & von Wirth, 2016: 9, figs 1, 2a–c, 3a–f, 4a–d.

*Phlogiellusbaeri* West, Nunn & Hogg, 2012: 25, fig. 34.

*Phlogiellusbaeri* Nunn, West & von Wirth, 2016: 12, figs 5a, b, 6a–f, 7a–g, 8a–e.

*Phlogiellusbirulai* Bariev & Logunov, in Bariev, Logunov & Son, 2024: 584, figs 1–14.

*Phlogiellusbogadeki* Nunn, West & von Wirth, 2016: 19, figs 10, 11a–e, 12a–d, 13a–c.

*Phlogiellusbundokalbo* Barrion & Litsinger, 1995: 22, fig. 5a–q.

*Phlogiellusdaweiensis* Sivayyapram & Warrit, in Sivayyapram et al. 2020: 490, figs 1a, b, 2a, b, 3a, b, 4a–d, 5a–c, 6a, b, 7a, b, 8a–d, 9a–c.

*Phlogiellusinermis* Giltay, 1934: 2, fig. 1b.

*Phlogiellusinsulanus* Hirst, 1909: 385, pl. 24, fig. 5.

*Phlogiellusinsulanusborneoensis* Schmidt, 2015d: 51, figs 3–5.

*Phlogiellusjiaxiangi*Lin et al. 2021b : 131, figs 34a–c, 35a–g, 36a–g, 37a–e.

*Phlogiellusjohnreylazoi* Nunn, West & von Wirth, 2016: 24, figs 18a, b, 19a–f, 20a–f, 21a–d, 22a–d, 23a–c.

*Phlogiellusmoniqueverdezae* Nunn, West & von Wirth, 2016: 33, figs 25a, b, 26a–f, 27a–e, 28a–f, 29a–c.

*Phlogiellusobscurus* Nunn, West & von Wirth, 2016: 37, figs 31a, b, 32a–d, 33a–e, 34c.

*Phlogiellusorophilus* Nunn, West & von Wirth, 2016: 38, figs 35a–d, 36a–d, 37a–c.

*Phlogielluspelidnus* Nunn, West & von Wirth, 2016: 38, figs 38, 39a–f, 40a–d, 41a–f, 42a–e.

*Phlogiellusquanyui*Lin et al. 2021a: 289, figs 1a–c, 2a–g, 3a–g.

*Phlogiellusraveni* Sivayyapram & Warrit, in Sivayyapram et al. 2020: 498, figs 10a, b, 11a, b, 12a, b, 13a–d, 14a–c, 15a, b, 16a, b, 17a–d, 18.

*Phlogielluswatasei* Zhu & Zhang, 2008: 444, figs. 9a–i.

*Phlogiellusxinping* Zhu & Zhang, 2008: 440, figs. 8a–i.

The comparison of illustrations of the spermathecae in females and male palps of the 12 *Phlogiellus* species within the *Yamia* group are given in Suppl. material 1: Fig. S1, Table S1.

## ﻿Taxonomy

### ﻿Mygalomorphae Pocock, 1892

**Theraphosidae Thorell, 1869**

**Selenocosmiinae Simon, 1889**

***Phlogiellus* Pocock, 1897**

***Yamia* Kishida, 1920 (syn. by West et al. 2012: 33)**

***Baccallbrapo* Barrion & Litsinger, 1995 (syn. by Haupt and Schmidt 2004: 220)**

#### ﻿ Phlogiellus khampheng

Taxon classificationAnimaliaAraneaeTheraphosidae

Sriranan, Songsangchote & Chomphuphuang sp. nov.

07076CE3-0077-51C1-BDE2-1D76B2030376

https://zoobank.org/B4A410EB-D1C9-4B4F-BB62-C448CA78E9C9

Figs 1 , 2 , 3 , 4 , 5 , 6 , 7 , 8A, B , 9

##### Type material.

**Lao PDR**: ***Holotype*** • ♂ (NUoL00058–PKP0001). ***Paratypes*** • 1 ♂ (NUoL00058–PKP0002), • 4 ♀ (NUoL00058–PKP0003, NUoL00058–PKP0004, NUoL00058–PKP0005, NUoL00058–PKP0006), deposited at NUOL, **Lao PDR: Pakse**: Champasack Province (15°05'36.3"N, 105°48'56.2"E), elevation 265 m, 23 Aug. 2023, Patipan Sriranan, Chaowalit Songsangchote, Odeth Sihavong, Phoukhanh Sayavongsa, Keolamphanh Sidavong, Lilammone Satakoun, Wuttikrai Khaikaew, Paveen Piyatrakulchai and Narin Chomphuphuang leg.

**Figures 2.**
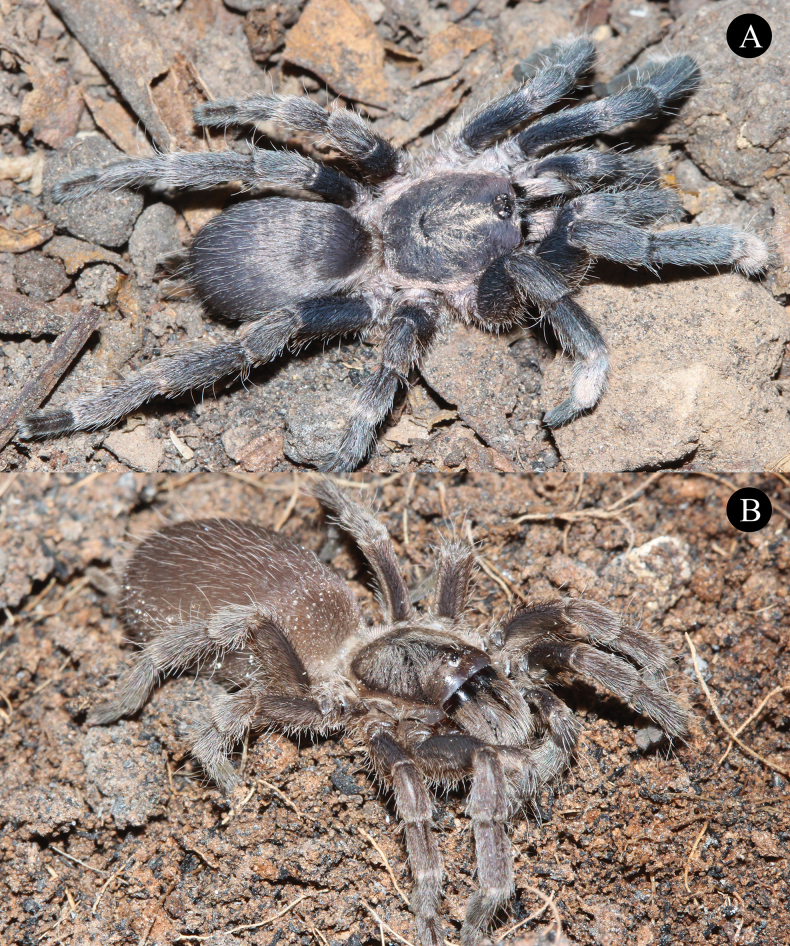
*Phlogielluskhampheng* sp. nov. **A.** Holotype ♂, NUoL00058–PKP0001; **B.** Paratype ♀, NUoL00058–PKP0003.

##### Diagnosis.

*Phlogielluskhampheng* sp. nov. was included in *Phlogiellus* based on the presence of a strong single retrolateral keel on the male embolus and a third claw on leg IV. *P.khampheng* sp. nov. is classified in the *Yamia* group (Kishida 1920) of *Phlogiellus*, similar to *P.aper*, *P.birulai*, *P.brevipes*, *P.bundokalbo*, *P.daweiensis*, *P.longipalpus*, *P.moniqueverdezae*, *P.mutus*, *P.quanyui*, *P.raveni*, and *P.watasei* due to the absence of maxillary lyra in female specimens. The *P.khampheng* sp. nov. differs from *P.aper*, *P.birulai*, *P.brevipes*, *P.daweiensis*, *P.longipalpus*, *P.mutus*, and *P.watasei* in having all metatarsal scopulae undivided, and differs from *P.raveni* and female *P.bundokalbo* in tarsal scopula division (divided on tarsus II, III and IV). The male *P.khampheng* sp. nov. differs from *P.brevipes*, *P.daweiensis*, *P.moniqueverdezae*, *P.quanyui*, *P.raveni*, and *P.watasei*, except *P.longipalpus*, by having a longer and more slender embolus with a distinct curve (Fig. 8). It can be further distinguished from *P.moniqueverdezae* by the narrower width at the base of the embolus. *P.khampheng* sp. nov. can also be distinguished from *P.moniqueverdezae* by geographical distribution with *P.moniqueverdezae* being found nearly 1,000 km away across the Gulf of Thailand. Furthermore, the male *P.khampheng* sp. nov. can be distinguished by the angle between the lowest and highest point of the embolus (ALH), which is 59°, compared to 77° in *P.longipalpus* and 44° in *P.moniqueverdezae* (Fig. 8). The female of *P.khampheng* sp. nov. differs from all other species in the *Yamia* group in the shape of the female spermathecae (Except female *P.aper* does not have described) which are twin receptacles with sub-apical buds (Fig. 6).

##### Description.

**Figures 3.**
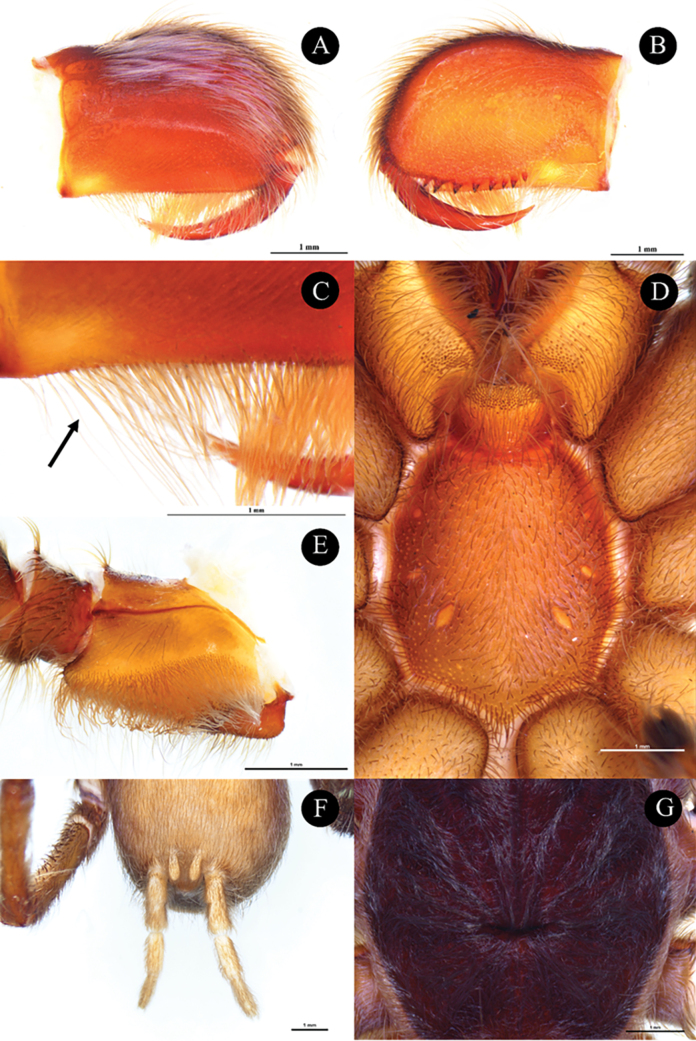
*Phlogielluskhampheng* sp. nov. Holotype, ♂, NUoL00058–PKP0001. **A.** Chelicerae, retrolateral view; **B.** Chelicerae, prolateral view; **C.** Chelicerae striker, retrolateral view arrowed; **D.** Sternum, ventral view; **E.** Right maxilla, prolateral view; **F.** Spinneret, ventral view, **G.** Foveal groove, dorsal view. Scale bars: 1 mm.

**Male. *Holotype*** ♂ NUoL00058–PKP0001: Color dark brown in life (Fig. 2A). Total length 16.89 (including chelicerae); carapace 5.23 width, 6.29 length, 1.77 high; procurved deep fovea (Fig. 3G), 0.98 width; carapace dark brown, with cover of short, grayish white hairs dorsally, chocolate brown on lateral margins (Fig. 2A), Ocular tubercle 1.22 width, 0.75 length, Clypeus absent. PER slightly recurved and AER slightly procurved; eyes whitish, ALE larger than the round AME; AME 0.20 length 0.19 width; ALE 0.32 length 0.19 width; PLE 0.27 length 0.17 width; PME 0.21 length 0.15 width; eye interdistances: PME–PME 0.57; PME–PLE 0.04; PLE–PLE 0.83; ALE–PLE 0.13; ALE–PME 0.20; ALE–ALE 0.70; AME–PME 0.14; AME–AME 0.20; AME–ALE 0.08; and AME–PLE 0.27. Chelicerae 3.23 length, 2.18 width, dark brown with eight teeth (Fig. 3A, B), three horizontal rows with a series of needle form striker <40 (Fig. 3C), Labium dark brown, 1.17 width, 0.84 length with 211 cuspules. Maxilla dark brown, 1.13 width, 2.16 length with 114 cuspules, covered with orange setae on prolateral surface and maxillary lyra absent (Fig. 3E). Sternum dark brown, 2.70 width, 3.11 length with soft white hairs and strong dark hairs, with 3 pairs of sigillae present near lateral margin of coxa I, II, and III (Fig. 3D) . Anterior pair 0.16 from sternal margin 0.07 width, 0.11 length; median pair 0.19 from sternal margin 0.10 width, 0.20 length; posterior pair 0.40 from sternal margin 0.17 width, 0.40 length.

**Figure 4.**
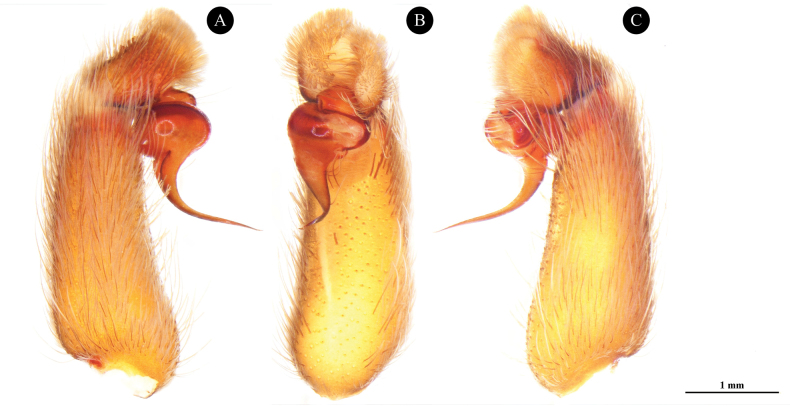
*Phlogielluskhampheng* sp. nov. Holotype, ♂, NUoL00058–PKP0001 right palp. **A.** Retrolateral view; **B.** Ventral view; **C.** Prolateral view.

***Abdomen*** 5.06 width, 7.93 length dark brown covered with short dark brown and long grayish white hairs dorsally, ventrally, and laterally. Spinnerets dark brown, covered with dark brown, thin and long hairs (Fig. 3F) ; PMS 0.83 length, 0.30 width; PLS 3.74 length basal segment, median segment and apical segment (1.44, + 1.16, + 1.14), width (0.49, + 0.44, + 0.40). ***Legs*** dark brown, retrolateral and prolateral of femur covered with dark hair. coxa, trochanter, patella and tibia dark brown and covered with dark brown hairs, metatarsus dark brown, metatarsus leg I and II covered with short and long grayish white hairs (in apical tibia) (Fig. 2A). Spination: metatarsus III ventral 0–0–1 (apical), metatarsus III prolateral 0–0–3 (apical), metatarsus III retrolateral 0–0–1 (apical), metatarsus IV ventral 0–0–1 (apical), metatarsus IV prolateral 0–0–3 (apical), metatarsus IV retrolateral 0–0–1 (apical), Length of leg and palp segment show in Table 1, tibial apophysis absent. Tarsal I, II, III with two claws and tarsus IV with three claws, teeth on claws absent. Scopula undivided on metatarsus. Scopula completely divided by row of long spines on tarsus III and IV, undivided on tarsus I and II. Pedipalps dark brown, covered with long and short grayish white hairs on tibia, two lobes dark brown scopula on cymbium, embolus and bulb light brown (Fig. 4).

**Table 1.** Legs and palp measurements (in mm) of holotype ♂ NUoL00058–PKP0001 *Phlogielluskhampheng* sp. nov.

	I	II	III	IV	Palp
Fem	5.31	4.06	3.69	4.92	2.99
Par	3.30	2.30	2.02	2.44	1.61
Tib	3.98	3.51	2.27	3.92	2.93
Met	2.53	2.17	2.37	4.35	–
Tar	2.16	2.16	1.72	1.93	0.85
Total	17.28	14.20	12.07	17.56	8.38

***Palp bulb and embolus*** (PBL+ELS) 1.82 length, palp bulb oval shape and partly concave, 0.90 width (PBW), 0.65 length (PBL). Embolus needle-like shape 0.42 width (EW), 1.17 length along a straight line with the bulb (ELS) and 1.33 embolus length along the curve (ELC), embolus thin, curve and twist on needle tip. Single longitudinal keel present (Fig. 4A). Palp bulb twisted at 59° angle between highest and lowest point of embolus (ALH) (Fig. 8). Ratios; ELC/PBL = 2.04, ELS/PBL = 1.8, EW/PBL = 0.64, ELC/EW = 3.16 and ELS/EW = 2.78.

**Figure 5.**
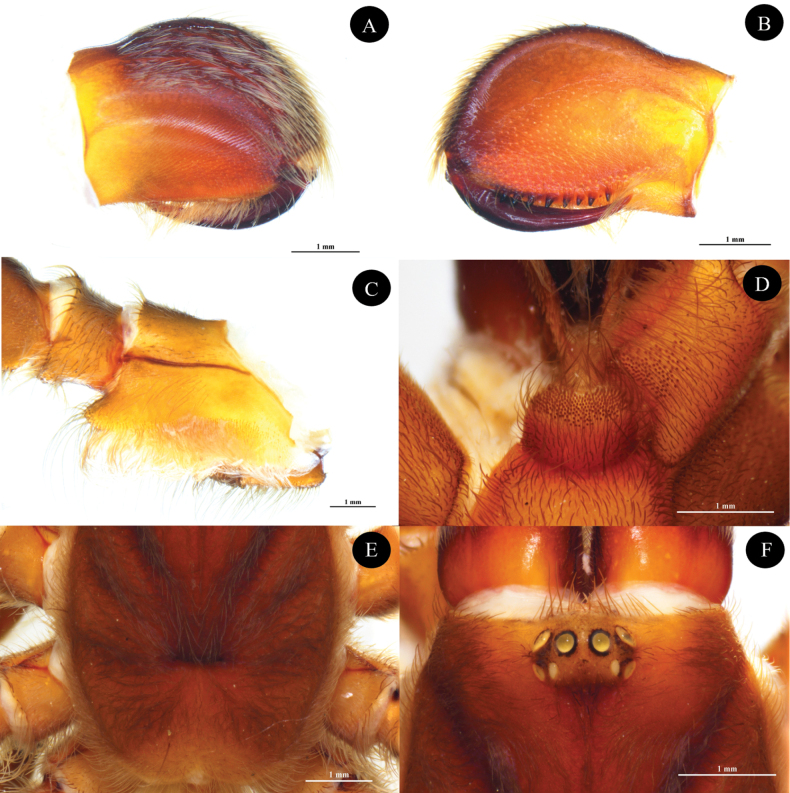
*Phlogielluskhampheng* sp. nov. **A–C.** Paratype ♀, NUoL00058–PKP0003; **D–F.** Paratype ♀, NUoL00058–PKP0004. **A.** Chelicerae, retrolateral view; **B.** Chelicerae, prolateral view; **C.** Right maxilla, prolateral view; **D.** Maxilla, labium and cuspules, ventral view; **E.** Foveal groove, dorsal view; **F.** Ocular tubercle. Scale bars: 1 mm.

**Female. *Paratype*** ♀, NUoL00058–PKP0003: Color chocolate brown in life, carapace brown (Fig. 2B). Total length 20.92 (including chelicerae); carapace 4.42 width, 6.70 length, 2.76 high; procurved deep foveal, 1.10 width; carapace brown with a cover of short, whitish hairs dorsally, chocolate brown on lateral margins (Fig. 2B). Ocular tubercle 1.15 width, 0.86 length, Clypeus absent. PER slightly recurved; AER slightly procurved; eyes whitish, ALE larger than the round AME; AME 0.20 length 0.21 width; ALE 0.31 length 0.16 width; PLE 0.22 length 0.15 width; PME .0.24 length 0.13 width; eye interdistances: PME–PME 0.52; PME–PLE 0.05; PLE–PLE 0.93; ALE–PLE 0.12; ALE–PME 0.15; ALE–ALE 0.66; AME–PME 0.10; AME–AME 0.11; and AME–ALE 0.05. Chelicerae 3.48 length, 270 width, dark brown with nine teeth, three horizontal rows with a series of needle form striker <40 (Fig. 5A, B). Labium dark brown, 1.33 width, 0.91 length with 260 cuspules. Maxilla dark brown, 1.36 width, 2.22 length with 133 cuspules, covered with orange setae on prolateral surface and maxillary lyra absent. (Fig. 5C). Sternum dark brown, 2.72 width, 3.21 length with soft white hairs and strong dark hairs, with 3 pairs of sigillae present near lateral margin of coxa I, II and III, anterior pair 0.81 from sternal margin 0.09 width, 0.11 length; median pair 0.20 from sternal margin 0.10 width, 0.20 length; posterior pair 0.50 from sternal margin 0.14 width, 0.31 length.

**Figure 6.**
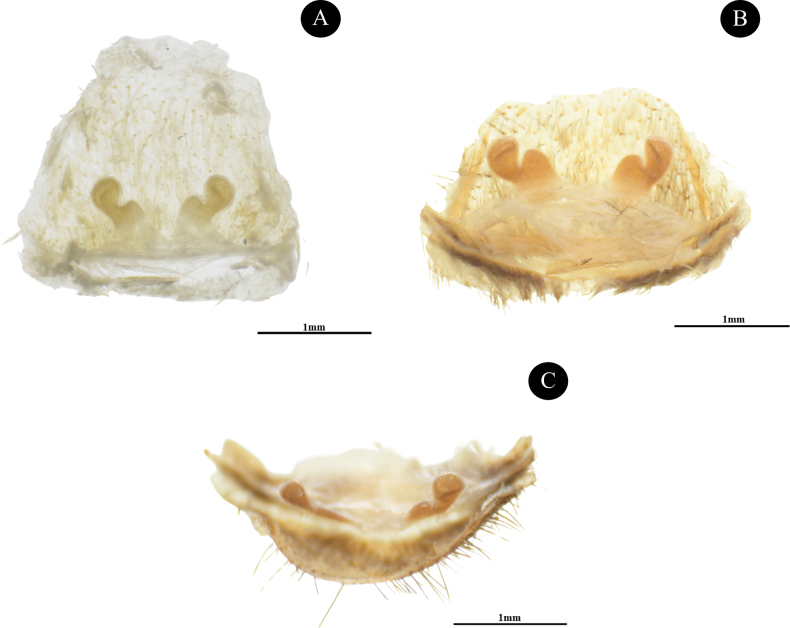
*Phlogielluskhampheng* sp. nov. **A.** Paratype ♀, NUoL00058–PKP0004: **B, C.** Paratype ♀, NUoL00058–KP0005. **A, B.** Spermathecae, dorsal view; **C.** Spermathecae, apical view. Scale bars: 1 mm.

***Abdomen*** 6.05 width, 10.13 length, dark brown covered with short dark brown and long grayish white hairs dorsally, ventrally, and laterally. Spinnerets dark brown, covered with dark brown longer and thinner hairs; PMS 0.68 length, 0.34 width; PLS 2.80 length basal segment, median segment and apical segment (0.83, + 0.91, + 1.06), width (0.49, + 0.50, + 0.41).

**Figure 7.**
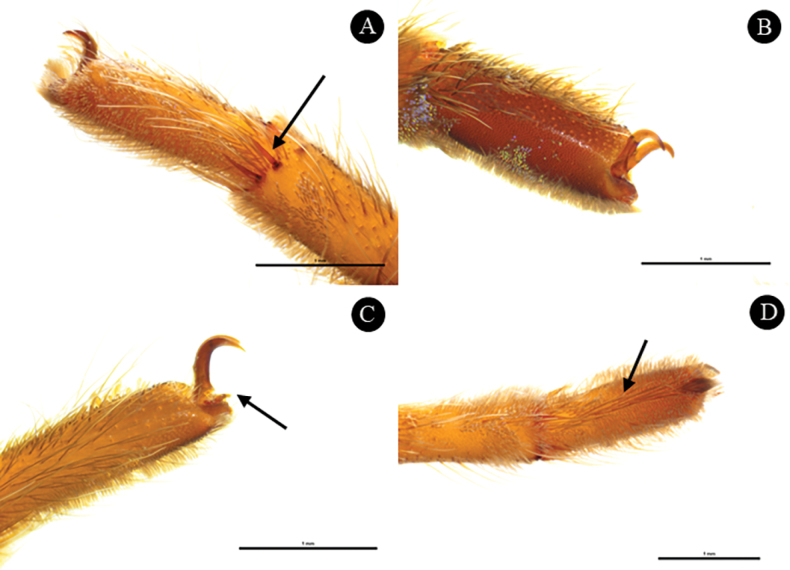
*Phlogielluskhampheng* sp. nov. Paratype ♀, NUoL00058–PKP0003. **A.** Right tarsus III, prolateral view, arrow indicates spine on metatarsus; **B.** Right tarsus I, retrolateral view, claws of tarsus; **C.** Right tarsus IV, retrolateral view, arrow indicates third claws; **D.** Right tarsus III, ventral view, scopula divided, arrow indicates row of long spines Scale bars: 1 mm.

***Legs*** dark brown, retrolateral and prolateral sides of femur covered with dark hair. Coxa and trochanter dark brown and covered with dark brown hairs. Patella, tibia, and metatarsus dark brown and covered with short and long brownish white hairs (Fig. 2B) Spination: metatarsus III ventral 0–0–1 (apical), metatarsus III prolateral 0–0–3 (apical) (Fig. 7A), metatarsus III retrolateral 0–0–1 (apical), metatarsus IV ventral 0–0–1 (apical), metatarsus IV prolateral 0–0–3 (apical), metatarsus IV retrolateral 0–0–1 (apical). Length of leg and palp segment show in Table 2. Tarsus I, II, III with two claws (Fig. 7B) and tarsus IV with three claws, teeth on claws absent (Fig. 7C). Scopula undivided on metatarsus. Scopula completely divided on tarsus II, III and IV, undivided on tarsus I. Scopula divided by row of long spines. (Fig. 7D).

**Table 2.** Legs and palp measurements (in mm) of paratype ♀, NUoL00058–PKP0003 *Phlogielluskhampheng* sp. nov.

	I	II	III	IV	Palp
Fem	4.57	3.47	3.19	4.41	3.14
Par	2.68	2.11	1.85	2.15	1.89
Tib	3.28	2.69	1.82	3.21	1.84
Met	2.23	1.82	2.10	3.21	–
Tar	1.71	1.65	1.58	1.81	1.88
Total	14.42	11.74	10.54	14.79	8.75

**Figure 8.**
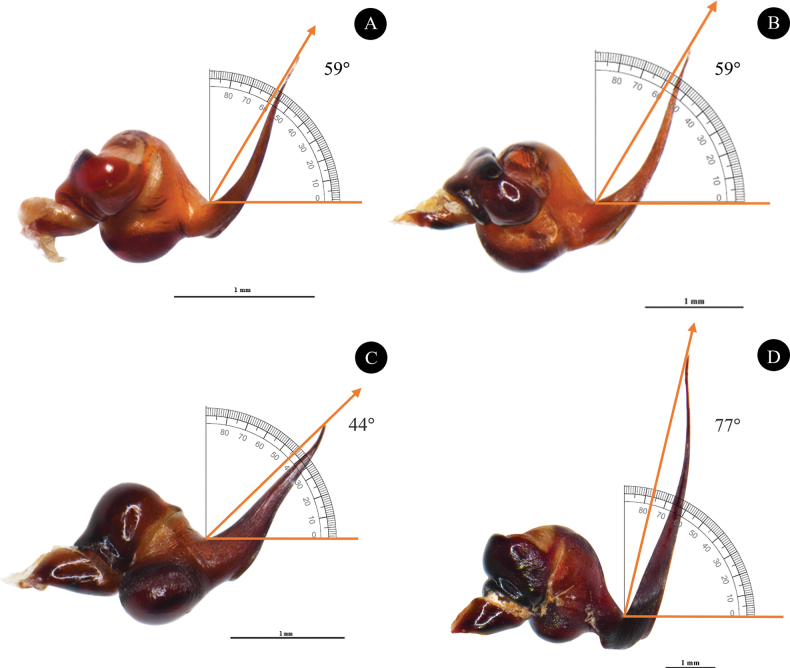
Angle of the lowest to highest point of male embolus (ALH). **A.***Phlogielluskhampheng* sp. nov. holotype NUoL00058–PKP0001 ♂, prolateral view; **B.***Phlogielluskhampheng* sp. nov. paratype NUoL00058–PKP0002 ♂, prolateral view; **C.***Phlogiellusmoniqueverdezae* ♂ Non-type C6–VA1, prolateral view; **D.***Phlogielluslongipalpus* ♂ Non-type B1–NA1, prolateral view. Scale bars: 1 mm.

***Spermatheca*** twin receptacles with sub-apical buds present; basal 0.44 width, 0.43 high, sub-apical bud 0.33 width, 0.73 high; the tops of sub-apical buds are ridged and swollen.

**Female. *Paratype*** ♀, NUoL00058–PKP0004: Color chocolate brown (in life), carapace brown. Total length 20.46 (including chelicerae); carapace 4.12 width, 5.58 length, 2.12 high; procurved deep fovea, 0.75 width (Fig. 5E); carapace brown with a cover of short, whitish hairs dorsally, chocolate-brown on lateral margins. Ocular tubercle 1.07 width, 0.67 length. (Fig. 5F). PER slightly recurved and AER slightly procurved; eyes whitish, ALE larger than the round AME; AME 0.19 length 0.18 width; ALE 0.27 length 0.13 width; PLE 0.18 length 0.10 width; PME 0.17 length 0.11 width; eye interdistances: PME–PME 0.52; PME–PLE 0.05; PLE–PLE 0.84; ALE–PLE 0.13; ALE–PME 0.18; ALE–ALE 0.57; AME–PME 0.13; AME–AME 0.13; and AME–ALE 0.09. Chelicerae 2.89 length, 2.15 width, dark brown with nine teeth, three horizontal rows with a series of needle form striker <40, Labium dark brown, 1.14 width, 0.80 length with 229 cuspules (Fig. 5D). Maxilla dark brown, 1.21 width, 2.08 length with 109 cuspules, covered with orange setae on prolateral surface and maxillary lyra absent. Sternum dark brown, 2.43 width, 2.95 length with soft white hairs and strong dark hairs, with three pairs of sigillae present near lateral margin of coxa I, II, and III, anterior pair 0.13 from sternal margin 0.06 width, 0.11 length; median pair 0.20 from sternal margin 0.09 width, 0.18 length; posterior pair 0.40 from sternal margin 0.11 width, 0.27 length.

*Abdomen* dark brown 6.23 width, 10.60 length covered with short dark brown and long grayish white hirsute dorsally, ventrally, and laterally. Spinnerets dark brown, covered with dark brown longer and thinner hairs; PMS 0.73 length, 0.31 width; PLS 2.81 length basal segment, median segment and apical segment (0.85, + 0.90, + 1.06), width (0.49 + 0.53 + 0.44).

***Legs*** dark brown, retrolateral and prolateral of femur covered with dark hair. Coxa and trochanter dark brown and covered with dark brown hairs. Patella, tibia, and metatarsus dark brown and covered with short and long brownish white hairs. Spination: metatarsus III ventral 0–0–1 (apical), metatarsus III prolateral 0–0–3 (apical), metatarsus III retrolateral 0–0–1 (apical), metatarsus IV ventral 0–0–1 (apical), metatarsus IV prolateral 0–0–3 (apical), metatarsus IV retrolateral 0–0–1 (apical), Length of leg and palp segment show in Table 3. Tarsi I, II, III with two claws and tarsus IV with three claws, teeth on claws absent. Scopula undivided on metatarsus. Scopula completely divided on tarsus II, III, and IV, undivided on tarsus I. Scopula divided by row of long spines.

**Table 3.** Legs and palp measurements (in mm) of paratype ♀, NUoL00058–PKP0004 *Phlogielluskhampheng* sp. nov.

	I	II	III	IV	Palp
Fem	4.43	3.84	3.13	4.15	3.25
Par	2.77	2.27	1.88	2.31	1.93
Tib	3.06	2.56	1.72	3.26	2.14
Met	2.12	1.90	1.74	2.86	–
Tar	1.88	1.73	1.53	1.68	2.08
Total	14.26	12.30	10.00	14.06	9.40

***Spermatheca*** twin receptacles with sub-apical buds present (Fig. 6A); basal 0.30 width, 0.42 high, sub-apical bud 0.25 width, 0.74 high, the tops of sub-apical buds are ridged and swollen (Fig. 6C).

##### Distribution and natural history.

**Figure 9.**
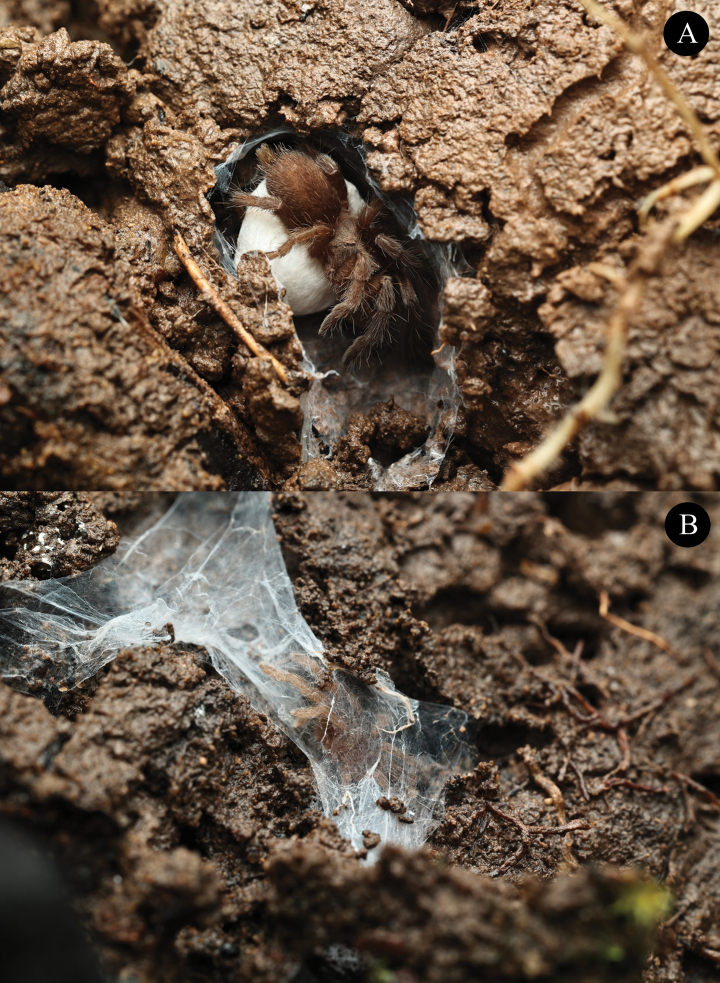
*Phlogielluskhampheng* sp. nov. **A.** Non-type NUoL00058–PKP0006 ♀ adult carrying an egg sac; **B.** Subterranean system of silk tube retreats under rock.

*Phlogielluskhampheng* sp. nov. specimens were collected from a mixed deciduous forest in the mountains near Pakse, Lao PDR. The habitat, situated at an elevation of 265 m, is characterized by a relatively open area with numerous boulders and cobbles. Large trees providing ample shade dominate the landscape. The spiders are opportunistic utilizing various microhabitats such as soil walls, under rocks (Fig. 9B), beneath timber, within cracks in timber (Fig. 1E), and inside tree hollows. Some nests are also constructed in cavities under bamboo roots (Fig. 1D). A silk structure is often built around the entrance of their nests. In this environment, *P.khampheng* sp. nov. is frequently found near colonies of ants and termites. Remains of these insects are often discovered within their retreats, suggesting that ants and termites form a significant part of their diet. During the observation, a notably small female *P.khampheng* sp. nov. (non-type NUoL00058–PKP0006 ♀) was encountered, measuring only 9.94 mm in total length from chelicerae to abdomen. This specimen was less than half the size of two paratypes: NUoL00058–PKP0004 (20.46 mm) and NUoL00058–PKP0003 (20.92 mm). Despite its diminutive size, this female was observed carrying an egg sac. Upon examination, the egg sac revealed a relatively low fecundity, containing only seven spiderlings (Fig. 9A) possibly due to age, with larger older females producing more young.

##### Etymology.

The species name “Khampheng” originates from the Lao and Thai languages, particularly in the Northeastern region, where it is used as a term of endearment to refer to someone who is cherished and precious to the speaker. The word carries a strong connotation of deep affection and high esteem, and it is often used in a loving and respectful manner when addressing or describing a person of great importance in one’s life. By choosing this name, the authors sought to convey the special and valuable relationship between Thailand and Laos, the two countries that collaborated closely in the discovery of this remarkable new tarantula species. “Khampheng” symbolizes the mutual respect, friendship, and cooperation that enabled the two nations to work together in advancing our understanding of the natural world and the incredible biodiversity it contains.

### ﻿Molecular phylogeny and species delimitation

**Figure 10.**
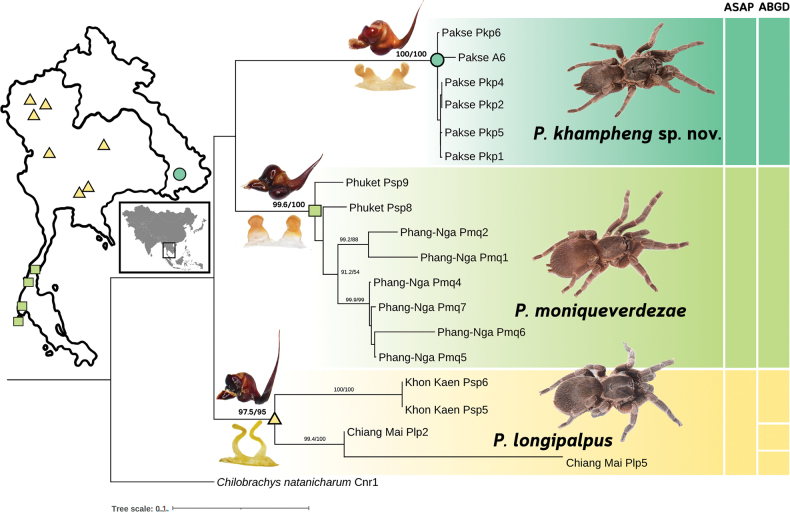
Maximum Likelihood (ML) phylogenetic tree constructed from COI sequence data of *Phlogiellus* species. The tree includes *P.khampheng* sp. nov. from Pakse (Laos), *P.moniqueverdezae* from Phuket and Phang-Nga (Thailand), and *P.longipalpus* from Khon Kaen and Chiang Mai (Thailand), accompanied by a species distribution locality map (left). Species are differentiated by distinct colours for clear visualization, with morphological features of reproductive organs used for species identification and differentiation. Node support values are shown as SH-aLRT support and ultrafast bootstrap (UFBoot) values calculated using IQ-TREE; nodes without support values did not meet threshold for reporting SH-aLRT support and ultrafast bootstrap (UFBoot) values < 90. Adjacent coloured bars represent species delimitation results from two genetic distance-based methods: ABGD (Automated Barcode Gap Discovery) and ASAP (Assemble Species by Automatic Partitioning).

The alignment, comprising more than 980 base pairs, included 18 sequences from three species of *Phlogiellus*, with *Chilobrachysnatanicharum* (Selenocosmiinae) designated as the outgroup for rooting. Using ModelFinder and the Bayesian Information Criterion (BIC), the TIM2+F+G4 model was identified as the best fit for the data. The Maximum Likelihood (ML) tree topology (log-likelihood score = -4442.3305) revealed three well-supported clades of *Phlogiellus* species (Fig. 10): *P.khampheng* sp. nov. from Pakse, Laos; *P.moniqueverdezae* from Phuket and Phang-Nga, Thailand; and *P.longipalpus* from Khon Kaen and Chiang Mai, Thailand. These clades received strong support, with SH-aLRT and ultrafast bootstrap values exceeding 95%. The ML tree topology is highly concordant with morphological analyses traditionally used to identify and distinguish species (Fig. 10). Among the species delimitation methods evaluated, the ASAP method (Partition 5) showed the highest agreement with the morphological analyses. This partition, with an ASAP score of 5.50, P-value (rank) = 9.32e-01 (8), W (rank) = 4.09e-03 (3), and a threshold distance of 0.125998, identified three species: *P.khampheng* sp. nov., *P.moniqueverdezae*, and *P.longipalpus*. Similarly, the ABGD species delimitation method (Partition 7) provided comparable results to ASAP, with a prior maximal distance (P) of 2.15e-02 and a barcode gap distance of 0.106. However, unlike ASAP, ABGD split *P.longipalpus* into three groups: *P.longipalpus* from Khon Kaen, Thailand (Psp5 and Psp6) were grouped together, while *P.longipalpus* from Chiang Mai, Thailand (Plp2 and Plp5) were split into two separate groups. Both molecular and morphological analyses supported the recognition of three distinct species, with the ASAP method providing results most consistent with morphological findings. ABGD, however, produced a minor deviation by splitting *P.longipalpus* into multiple groups, thereby highlighting strong genetic differentiation within this species.

## ﻿Discussion

*Phlogiellus* is one of the genera within the Theraphosidae family that still has classification problems, primarily due to its complex taxonomic history and the lack of clear diagnostic features. This can be shown with the features used between Nunn et al. (2016) and those suggested in Sivayyapram et al. (2020) with each group proposing differing opinions as to which features were most useful. The genus has undergone several revisions, with changes in species combinations and diagnostic characters over time. *Phlogiellus* can be divided into two groups, the *Phlogiellus* group (Pocock 1897) and the *Yamia* group (Kishida 1920). The key distinguishing feature between these groups is the presence of lyra on the prolateral face of the maxilla in the *Phlogiellus* group, which is absent in the *Yamia* group. The classification of Selenocosmiinae taxa lacking maxillary lyra, such as those in the *Yamia* group, poses a significant taxonomic challenge. One hypothesis suggests that these alyrate taxa could represent a sister lineage to all lyrate species. Alternatively, it has been proposed that *Yamia* may have lost its lyra secondarily and could therefore be more appropriately classified within *Phlogiellus* (as per Raven 2005). *Phlogielluskhampheng* sp. nov. belongs to the *Yamia* group due to the absence of the maxillary lyra. This characteristic is shared with several other species, including *P.aper*, *P.birulai*, *P.brevipes*, *P.bundokalbo*, *P.daweiensis*, *P.longipalpus*, *P.moniqueverdezae*, *P.mutus*, *P.raveni*, and *P.watasei*. However, *P.khampheng* sp. nov. differs from the other species in the *Yamia* group in several ways. The female’s spermathecae are twin receptacles with sub-apical buds that resemble those of *P.baeri* (Nunn et al. 2016: fig. 8b), but in *P.khampheng* sp. nov., these sub-apical buds are higher and larger. The male of *P.khampheng* sp. nov. also differs from *P.baeri* in the shape of the embolus that is narrower in width (EW) compared to that of *P.baeri* (West et al. 2012: fig. 34). Additionally, the male *P.khampheng* sp. nov. lacks a maxillary lyra, whereas it is present in male *P.baeri* (Nunn et al. 2016: fig. 6d).

According to Chomphuphuang et al. (2023), male palp characteristics are essential for describing species within the genus *Chilobrachys*. One key feature is the angle between the lowest and highest points of the embolus (ALH). In this study, the palp angle was measured in the male *P.khampheng* sp. nov., 59° (Fig. 8A, B), which is lower than the ALH of *P.longipalpus* at 77° (Fig. 8D) and higher than that of *P.moniqueverdezae* at 44° (Fig. 8C). These variations in ALH suggest that it could be a valuable tool for classifying species within *Phlogiellus*. However, to confirm its reliability as a diagnostic feature, it is necessary to conduct further measurements and DNA sequencing across additional specimens and species within the genus to substantiate its viability before this is used on a larger spectrum. This broader analysis would help establish whether the ALH consistently provides sufficient differentiation for accurate species identification.

Following Chomphuphuang et al. (2017), the pattern of scopula division has been highlighted as a distinguishing feature for mature specimens within the *Yamia* group, used by Raven (2005), Guadanucci (2005), and Nunn et al. (2016). In this study, we have revised and expanded upon previous works by incorporating all recent *Yamia* group species and adding data on the number of claws (Table 4). This information provides additional support for identifying species of *Phlogiellus* within the *Yamia* group. However, it cannot be applied to female *P.aper* (due to the lack of described specimens) or *P.brevipes* (which has not been examined). The results indicate that in *P.khampheng* sp. nov., tarsi I, II, and III each have two claws, while tarsus IV has three claws. This claw pattern is similar to that found in *P.daweiensis*, *P.longipalpus*, *P.moniqueverdezae*, *P.raveni*, and *P.watasei*. However, in *P.raveni*, this characteristic is observed only in females; males have three claws present on leg III (Table 4) . These findings highlight the potential of claw patterns as a useful taxonomic character within the *Phlogiellus* genus, while also underscoring the importance of considering sexual dimorphism in such traits.

**Table 4.** Scopula characteristics on metatarsi and tarsi I–IV of the *Yamia* group (Kishida 1920) in *Phlogiellus* lacking the maxillary lyra (Including *P.khampheng* sp. nov., *P.aper*, *P.brevipes*, *P.bundokalbo*, *P.daweiensis*, *P.longipalpus*, *P.moniqueverdezae*, *P.mutus*, *P.raveni*, *P.watasei*, and *P.birulai*). The table indicates whether the scopula are divided by a row of bristles or spines, or if they are absent (scopula: 🗶 = undivided, ✔ = divided, – = absent), as well as the number of claws.

Species	Metatarsus				Tarsus				Claws				Reference
	I	II	III	IV	I	II	III	IV	I	II	III	IV	
*P.khampheng* sp. nov. (♂, ♀)	🗶	🗶	🗶	🗶	🗶	✔	✔	✔	2	2	2	3	This study
*P.moniqueverdezae* Nunn, West & von Wirth, 2016 (♂, ♀)	🗶	🗶	🗶	🗶	🗶	✔	✔	✔	2	2	2	3	Nunn et al. 2016
*P.longipalpus* Chomphuphuang, Smith, Wongvilas, Sivayyapram, Songsangchote & Warrit, 2017 (♂, ♀)	🗶	🗶	🗶	✔	🗶	🗶	✔	✔	2	2	2	3	Chomphuphuang et al. 2017
*P.mutus* (Giltay, 1935) (♀)	🗶	🗶	–	✔	🗶	🗶	✔	✔	N/A	N/A	N/A	N/A	Giltay 1935; Chomphuphuang et al. 2017
*P.aper* (Simon, 1891) (♂)	🗶	🗶	🗶	✔	🗶	🗶	✔	✔	N/A	N/A	N/A	N/A	Simon 1891; Chomphuphuang et al. 2017
*P.watasei* (Kishida, 1920) (♂)	🗶	🗶	🗶	✔	🗶	🗶	🗶	✔	2	2	2	3	Zhu and Zhang 2008
*P.watasei* (Kishida, 1920) (♀)	✔	✔	✔	✔	✔	✔	✔	✔	2	2	2	3	Zhu and Zhang 2008
*P.bundokalbo* (Barrion & Litsinger, 1995) (♂)	🗶	🗶	🗶	🗶	🗶	✔	✔	✔	2	2	2	3	(Barrion and Litsinger 1995); Chomphuphuang et al. 2017
*P.bundokalbo* (Barrion & Litsinger, 1995) (♀)	🗶	🗶	🗶	🗶	🗶	🗶	🗶	🗶	2	2	2	3	(Barrion and Litsinger 1995); Chomphuphuang et al. 2017
*P.raveni* Sivayyapram & Warrit, 2020 (♂)	🗶	🗶	🗶	🗶	🗶	🗶	✔	✔	2	2	3	3	Sivayyapram et al. 2020
*P.raveni* Sivayyapram & Warrit, 2020 (♀)	🗶	🗶	🗶	–	✔	🗶	–	–	2	2	2	3	Sivayyapram et al. 2020 Note: absence of scopula may be an artifact of old collection
*P.daweiensis* Sivayyapram & Warrit, 2020 (♂)	🗶	🗶	🗶	✔	🗶	🗶	🗶	✔	2	2	2	3	Sivayyapram et al. 2020
*P.daweiensis* Sivayyapram & Warrit, 2020 (♀)	🗶	🗶	🗶	✔	🗶	🗶	✔	✔	2	2	2	3	Sivayyapram et al. 2020
*P.birulai* Bariev & Logunov, 2024 (♀)	✔	✔	✔	✔	✔	✔	✔	✔	2	2	2	3	Bariev et al. 2024

The taxonomy and classification of the genus *Phlogiellus* have undergone several revisions regarding the number of labial cuspules, another key morphological feature used in species identification. West et al. (2012) initially proposed a range of 200–350 labial cuspules for the genus, which was later revised by Nunn et al. (2016) to 160–320. Our study, summarized in Table 5, corroborates this revised range based on original species descriptions and examination of type material. The observed minimum number of labial cuspules is found in *P.baeri* (>160), while the maximum is seen in *P.pelidnus* (>320). However, Sivayyapram et al. (2020) raised concerns about the precision of these counts, particularly for *P.pelidnus*. They argued that the use of “>320” is imprecise and potentially problematic for diagnostic purposes, emphasizing the need for more exact quantification in taxonomic descriptions. Furthermore, Sivayyapram et al. (2020) discussed sexual dimorphism in cuspule numbers within *Phlogiellus* species. For instance, *P.moniqueverdezae* males have more than 200 cuspules, whereas females have more than 300. Our comparison in Table 5 also reveals distinct sexual dimorphism, such as in *P.longipalpus* (males with 202 cuspules and females with 271). Additionally, we found considerable intra-sexual variation, such as in *P.jiaxiangi* females (195–283) and males (283). In our study of *Phlogielluskhampheng* sp. nov., we observed labial cuspule numbers between 211 and 260 (with an average of 233.3±24.7), aligning with the broader range of 160–320. Given the significant variability both between and within species, as well as between sexes, we conclude that while the range of 160–320 labial cuspules remains useful for genus-level descriptions, it may not be sufficiently reliable for species-level diagnoses. The high degree of variation in this trait complicates its use as a definitive diagnostic character at the species level. Therefore, we recommend using this feature cautiously and in conjunction with other morphological traits when describing or identifying *Phlogiellus* species. In the future, molecular studies will be required to provide better support for the taxonomic classification of this subfamily.

**Table 5.** Body size (mm) and number of cuspules in the Southeast Asian dwarf tarantula genus *Phlogiellus* based on original species descriptions or examinations of type material.

Species	Body size (mm)	Number of cuspules	Reference
*P.aper* (Simon, 1891) (♂)	N/A	N/A	Simon 1891
*P.atriceps* Pocock, 1897 (♂)	17.0	>220	Nunn et al. 2016
*P.atriceps* Pocock, 1897 (♀)	19.0	N/A	Pocock 1897
*P.baeri* (Simon, 1877) (♂)	18.65	>160	Nunn et al. 2016
*P.baeri* (Simon, 1877) (♀)	15	N/A	Simon 1877
*P.bicolor* Strand, 1911 (♀)	17.00	N/A	Strand 1911
*P.birulai* Bariev & Logunov, 2024 (♀)	11.50	200–220	Bariev et al. 2024
*P.bogadeki* Nunn, West & von Wirth, 2016 (♀)	17.67	>200	Nunn et al. 2016
*P.brevipes* (Thorell, 1897) (♂)	15.00	N/A	Thorell 1897
*P.brevipes* (Thorell, 1897) (♀)	18.00	N/A	Thorell 1897
*P.bundokalbo* (Barrion & Litsinger, 1995) (♂)	13.41	N/A	Barrion and Litsinger 1995
*P.bundokalbo* (Barrion & Litsinger, 1995) (♀)	13.41	N/A	Barrion and Litsinger 1995
*P.daweiensis* Sivayyapram & Warrit, 2020 (♂)	15.20–18.08	221–235	Sivayyapram et al. 2020
*P.daweiensis* Sivayyapram & Warrit, 2020 (♀)	18.08–22.08	231–316	Sivayyapram et al. 2020
*P.inermis* (Ausserer, 1871) (♂)	17.00	N/A	Giltay 1934
*P.inermis* (Ausserer, 1871) (♀)	23.00	N/A	Giltay 1934
*P.insulanus* (Hirst, 1909) (♂)	18.00	N/A	Hirst 1909
*P.insulanusborneoensis* (Schmidt, 2015) (♂)	24.00	N/A	Schmidt 2015
*P.insularis* (Simon, 1877) (♀)	15.2	N/A	Simon 1877
*P.jiaxiangi* Lin & Li, 2021 (♂)	10.18	283	Lin and Li 2021
*P.jiaxiangi* Lin & Li, 2021 (♀)	15.87	195–283	Lin and Li 2021
*P.johnreylazoi* Nunn, West & von Wirth, 2016 (♂)	37.3	>200	Nunn et al. 2016
*P.johnreylazoi* Nunn, West & von Wirth, 2016 (♀)	43.34	>200	Nunn et al. 2016
*P.khampheng* sp. nov. (♂)	16.89	211	This study
*P.khampheng* sp. nov. (♀)	9.94–20.92	229–260	This study
*P.longipalpus* Chomphuphuang, Smith, Wongvilas, Sivayyapram, Songsangchote & Warrit, 2017 (♂)	13.70–21.0	202	Chomphuphuang et al. 2017
*P.longipalpus* Chomphuphuang, Smith, Wongvilas, Sivayyapram, Songsangchote & Warrit, 2017 (♀)	14.30–26.75	271	Chomphuphuang et al. 2017
*P.moniqueverdezae* Nunn, West & von Wirth, 2016 (♂)	24.06	>300	Nunn et al. 2016
*P.moniqueverdezae* Nunn, West & von Wirth, 2016 (♀)	27.13	>200	Nunn et al. 2016
*P.mutus* (Giltay, 1935) (♀)	14	N/A	Giltay 1935
*P.nebulosus* (Rainbow, 1899) (♀)	12.4	N/A	Rainbow 1899
*P.obscurus* (Hirst, 1909) (♂)	26.5	N/A	Hirst 1909
*P.ornatus* (Thorell, 1897) (♀)	12.00	N/A	Thorell 1897
*P.orophilus* (Thorell, 1897) (♀)	14.00–15.00	N/A	Thorell 1897
*P.pelidnus* Nunn, West & von Wirth, 2016 (♂)	30.21	>294	Nunn et al. 2016
*P.pelidnus* Nunn, West & von Wirth, 2016 (♀)	46.37	>320	Nunn et al. 2016
*P.quanyui* Lin, Li & Chen, 2021 (♂)	12.18	309	Lin et al. 2021
*P.quanyui* Lin, Li & Chen, 2021(♀)	14.90	239	Lin et al. 2021
*P.raveni* Sivayyapram & Warrit, 2020 (♂)	15.68	N/A	Sivayyapram et al. 2020
*P.raveni* Sivayyapram & Warrit, 2020 (♀)	16.00	N/A	Sivayyapram et al. 2020
*P.subinermis* Giltay, 1934 (♂)	16	N/A	Giltay 1934
*P.subinermis* Giltay, 1934 (♀)	22.5	N/A	Giltay 1934
*P.watasei* (Kishida, 1920) (♂)	14.20–14.22	268	Zhu and Zhang 2008
*P.watasei* (Kishida, 1920) (♀)	18.81	316	Zhu and Zhang 2008
*P.xinping* (Zhu & Zhang, 2008) (♂)	15.40	309	Zhu and Zhang 2008
*P.xinping* (Zhu & Zhang, 2008) (♀)	18.00	283	Zhu and Zhang 2008

## Supplementary Material

XML Treatment for Phlogiellus khampheng

## ﻿Additional information

### Use of AI

No use of AI was reported.

### Author ORCIDs

Patipan Sriranan https://orcid.org/0009-0002-4995-2515

Chaowalit Songsangchote https://orcid.org/0000-0001-7689-5363

Khamla Inkhavilay https://orcid.org/0009-0007-1060-0885

Narin Chomphuphuang https://orcid.org/0000-0003-0738-3879

### Data availability

All of the data that support the findings of this study are available in the main text or Supplementary Information.

## Supplementary materials

Supplementary material 1 Supplemental figures and table Data type docx Explanation note **fig. S1. A–J.** Spermathecae of *Phlogiellus* species (*Yamia* group), excluding *P.aper* (due to the lack of described female specimens) and *P.brevipes* (as the characteristics of the spermathecae are not illustrated), dorsal view; **A.***P.khampheng* sp. nov. Paratype ♀, NUoL00058–KP0005; **B.***P.daweiensis* modified from Sivayyapram et al. (2020); **C.***P.watasei* modified from Zhu & Zhang (2008); **D.***P.raveni* modified from Sivayyapram et al. (2020); **E.***P.mutus* modified from Schmidt (2010); **F.***P.bundokalbo* modified from Schmidt (2010); **G.***P.moniqueverdezae* modified from Nunn et al. (2016); **H.***P.longipalpus* modified from Chomphuphuang et al. (2017); **I.***P.quanyui* modified from Lin et al. (2021); **J.***P.birulai* modified from Bariev et al. (2024). **fig. S2. A–I.** Male palps of described *Phlogiellus* species (*Yamia* group), excluding *P.birulai* and *P.mutus* (due to the lack of described male specimens) as well as *P.aper* and *P.brevipes* (since the characteristics of their spermathecae are not illustrated). **A.***P.khampheng* sp. nov. Holotype, ♂, NUoL00058–PKP0001; **B.***P.daweiensis* modified from Sivayyapram et al. (2020); **C.***P.watasei* modified from Zhu & Zhang (2008); **D.***P.raveni* modified from Sivayyapram et al. (2020); **E.***P.bundokalbo* modified from Schmidt (2010); **F.***P.moniqueverdezae* modified from Nunn et al. (2016); **G.***P.longipalpus* modified from Chomphuphuang et al. (2017); **H.***P.quanyui* modified from Lin et al. (2021); **I.***P.aper* Lectotype, ♂, AR4675. **table S1.** Comparison of illustrations of the spermathecae in females and male palps of 12 *Phlogiellus* species in the *Yamia* group. https://binary.pensoft.net/file/1379331This dataset is made available under the Open Database License (http://opendatacommons.org/licenses/odbl/1.0/). The Open Database License (ODbL) is a license agreement intended to allow users to freely share, modify, and use this Dataset while maintaining this same freedom for others, provided that the original source and author(s) are credited.Patipan Sriranan, Chaowalit Songsangchote, Odeth Sihavong, Phoukhanh Sayavongsa, Keolamphanh Sidavong, Lilammone Satakoun, Khamla Inkhavilay, Narin Chomphuphuang, Ray Gabriel
