# Supplementary material for: ﻿A new species of Southeast Asian dwarf tarantula in the genus Phlogiellus Pocock, 1897, from Lao PDR (Theraphosidae, Selenocosmiinae)
Source: Zookeys. 2025 Jul 22;1247:19–43. doi: 10.3897/zookeys.1247.155398 (PMC12344373; doi:10.3897/zookeys.1247.155398)
Supplement: Supplementary material 1 — Supplemental figures and table [file zookeys-1247-019_article-155398__-s001.docx]

Supplemental Figure


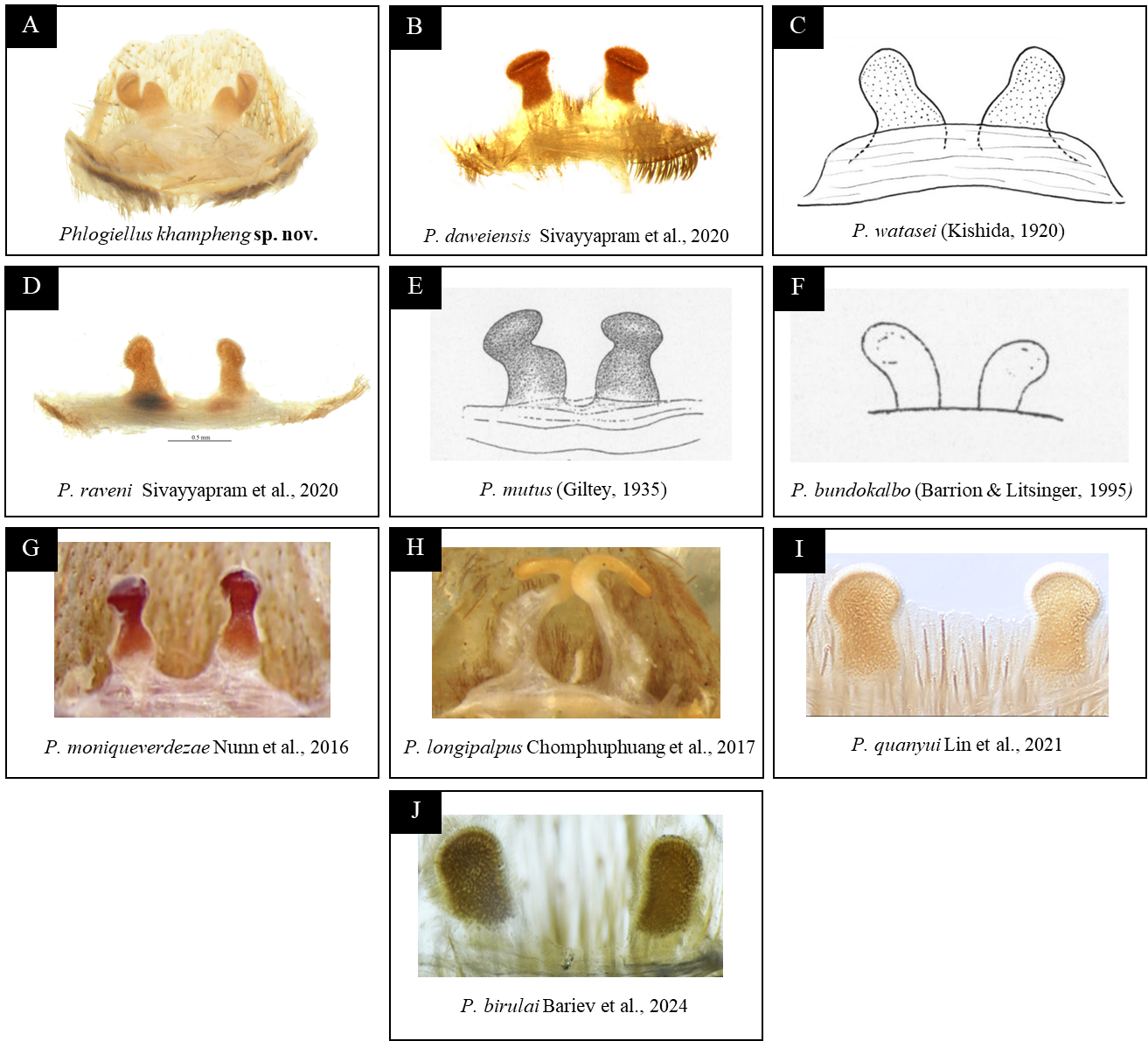


**Supplemental Figures1 A–J.** Spermathecae of *Phlogiellus* species (*Yamia* group), excluding *P. aper* (due to the lack of described female specimens) and *P. brevipes* (as the characteristics of the spermathecae are not illustrated), dorsal view **A** *P. khampheng* **sp. nov.** Paratype ♀, NUoL00058–KP0005, **B** *P. daweiensis* modified from Sivayyapram et al. (2020), **C** *P. watasei* modified from Zhu & Zhang (2008), **D** *P. raveni* modified from Sivayyapram et al. (2020), **E** *P. mutus* modified from Schmidt (2010), **F** *P. bundokalbo* modified from Schmidt (2010), **G** *P. moniqueverdezae* modified from Nunn et al. (2016), **H** *P. longipalpus* modified from Chomphuphuang et al. (2017), **I** *P. quanyui* modified from Lin et al. (2021), **J** *P. birulai* modified from Bariev et al. (2024).

**
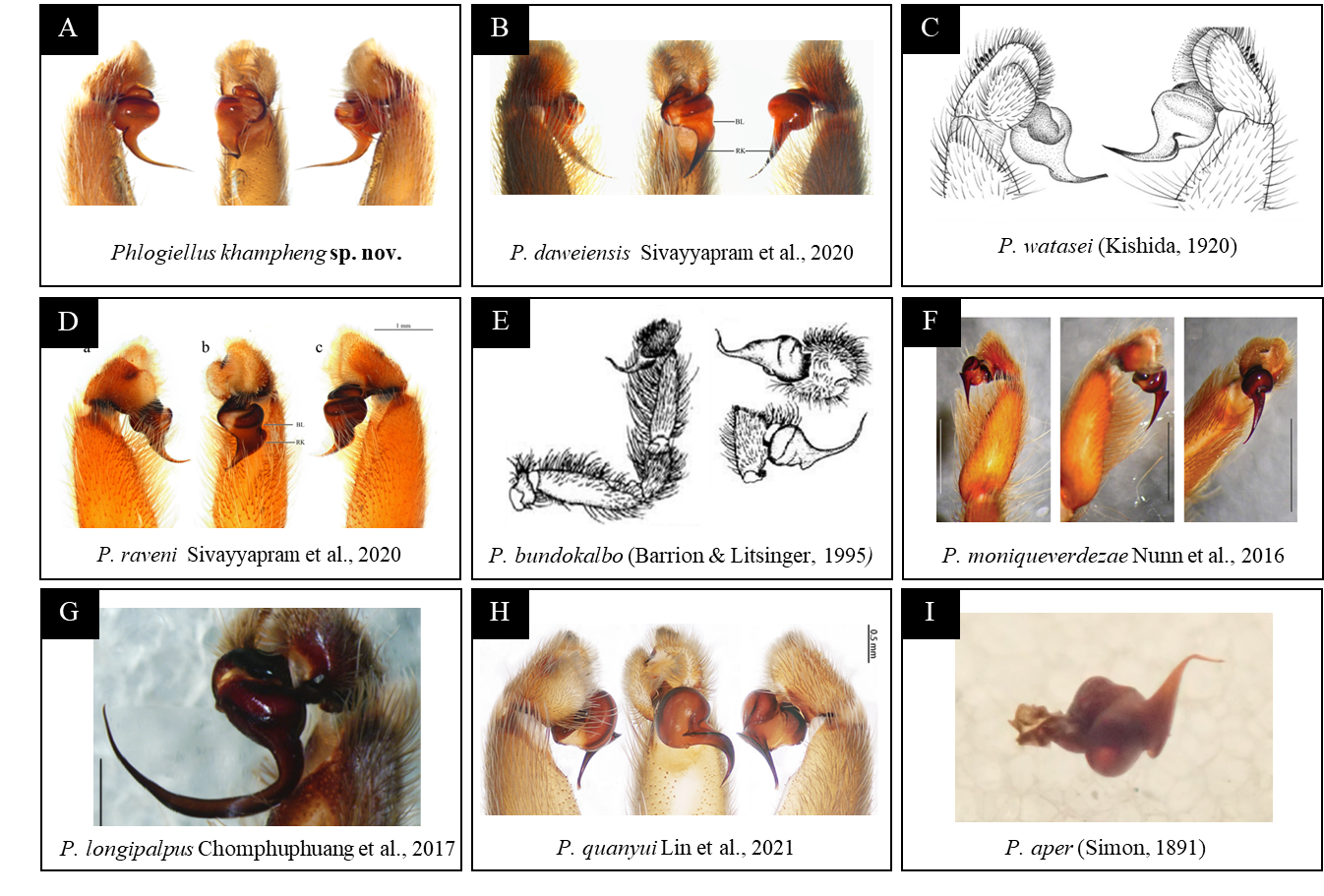
**

**Supplemental Figures2 A–I.** Male palps of described *Phlogiellus* species (*Yamia* group), excluding *P. birulai* and *P. mutus* (due to the lack of described male specimens) as well as *P. aper* and *P. brevipes* (since the characteristics of their spermathecae are not illustrated). **A** *P. khampheng* sp. nov. Holotype, ♂, NUoL00058–PKP0001, **B** *P. daweiensis* modified from Sivayyapram et al. (2020), **C** *P. watasei* modified from Zhu & Zhang (2008), **D** *P. raveni* modified from Sivayyapram et al. (2020), **E** *P. bundokalbo* modified from Schmidt (2010), **F** *P. moniqueverdezae* modified from Nunn et al. (2016), **G** *P. longipalpus* modified from Chomphuphuang et al. (2017), **H** *P. quanyui* modified from Lin et al. (2021), **I** *P. aper* Lectotype, ♂, AR4675.

**Table 1** Comparison of illustrations of the spermathecae in females and male palps of 12 *Phlogiellus* species in the *Yamia* group

| 1 | 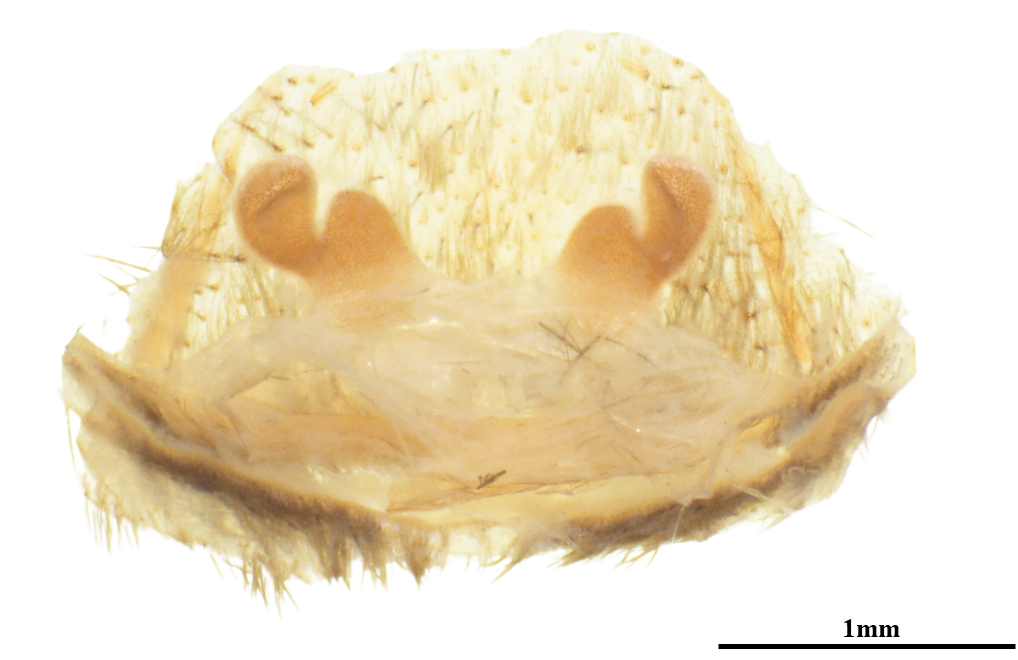  *P. khampheng* sp. nov. ♀ | 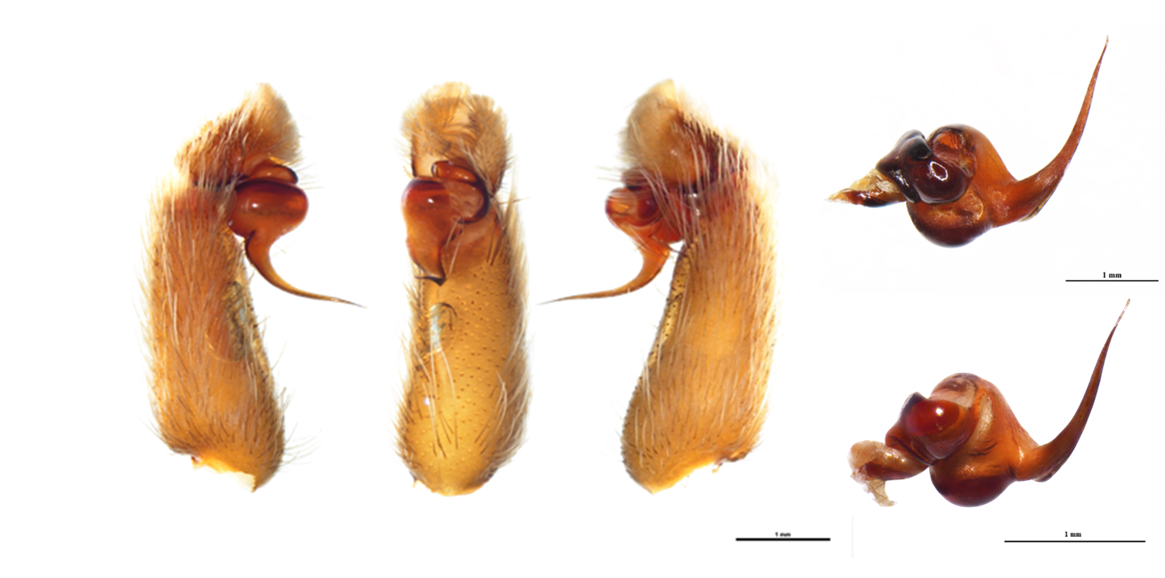  *P. khampheng* sp. nov. ♂ |
| --- | --- | --- |
| 2 | N/A  *Phlogiellus aper* (Simon, 1891) ♀ | 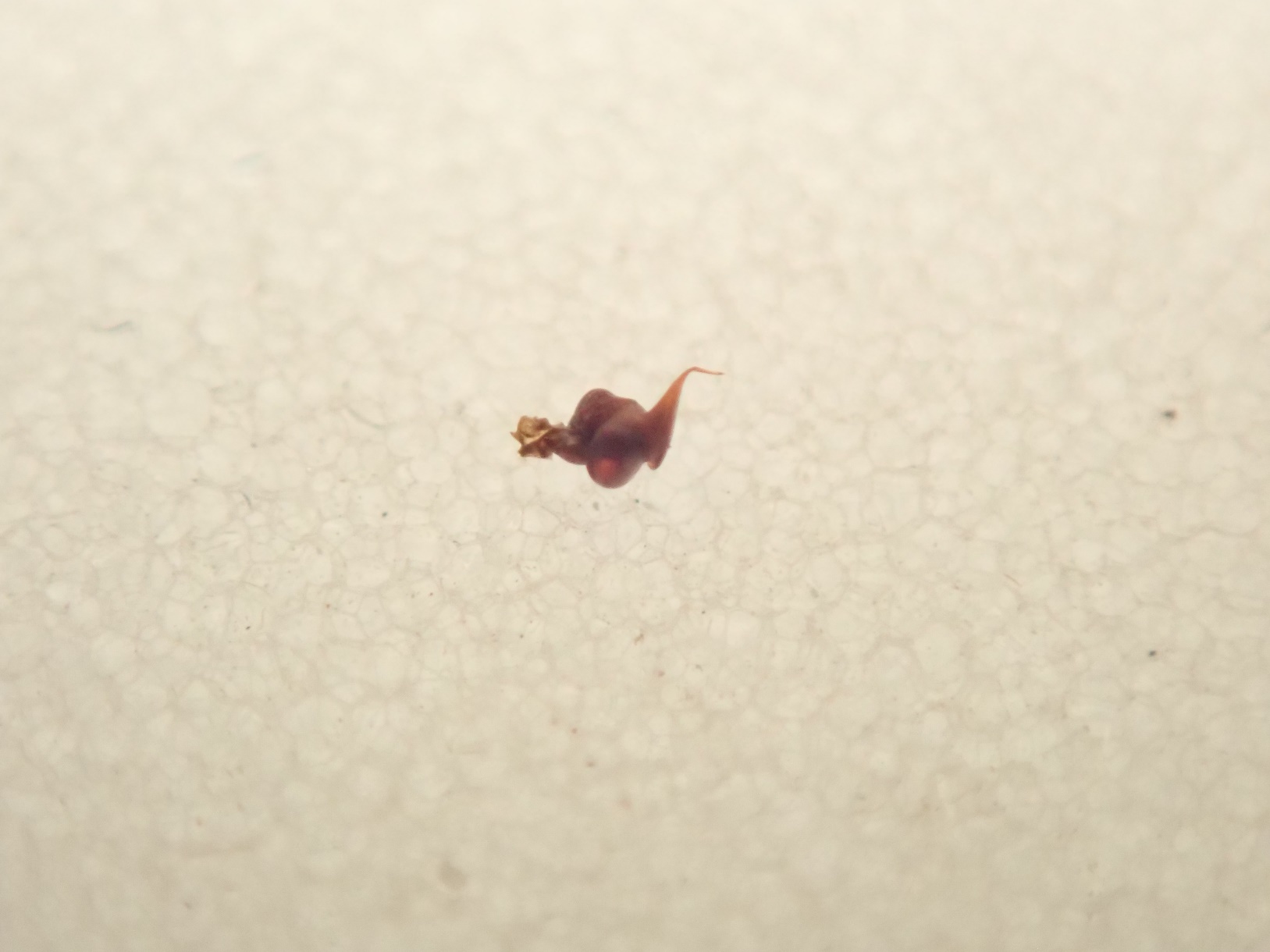  *Phlogiellus aper* (Simon, 1891) ♂ |
| 3 | *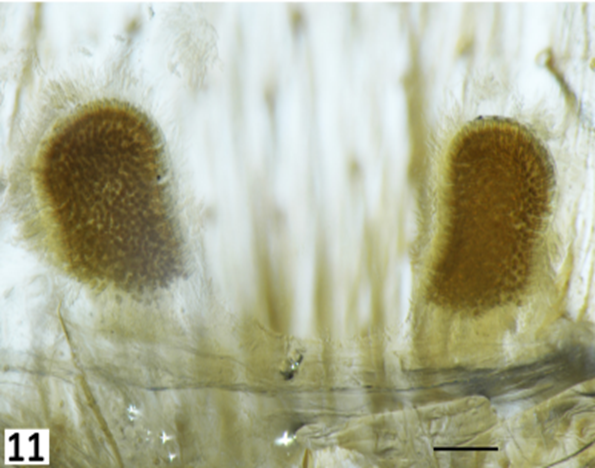*  *P. birulai* Bariev et al., 2024 ♀ | N/A  *P. birulai* Bariev et al., 2024 ♂ |
| 4 | N/A  *Phlogiellus brevipes* (Thorell, 1897) ♀ | N/A  *Phlogiellus brevipes* (Thorell, 1897) ♂ |
| 5 | *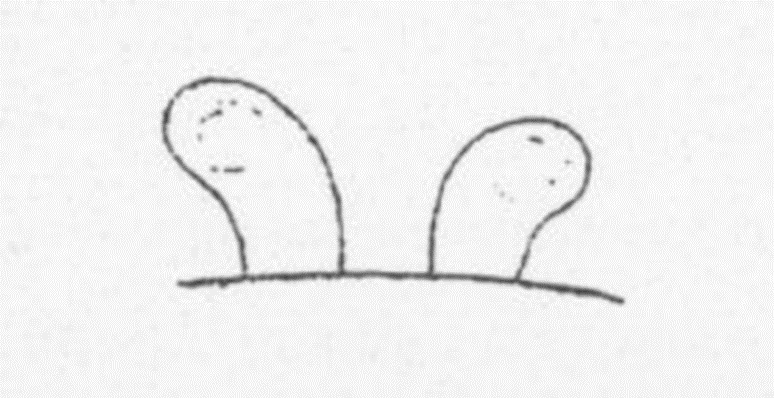*  *P. bundokalbo* (Barrion & Litsinger, 1995) ♀ | *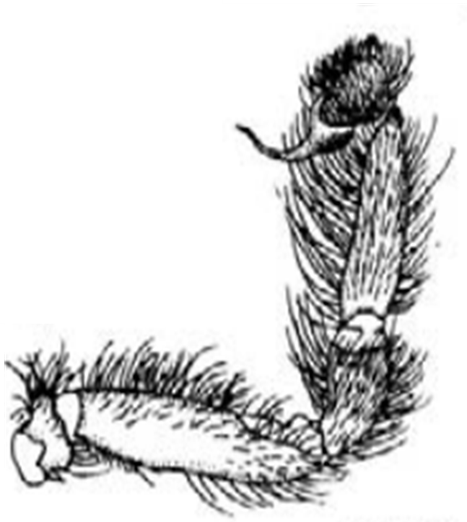* *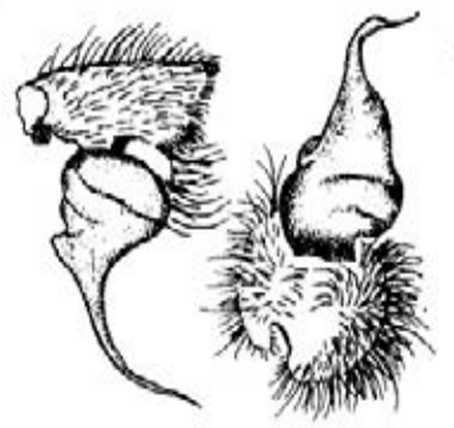*  *P. bundokalbo* (Barrion & Litsinger, 1995) ♂ |
| 6 | *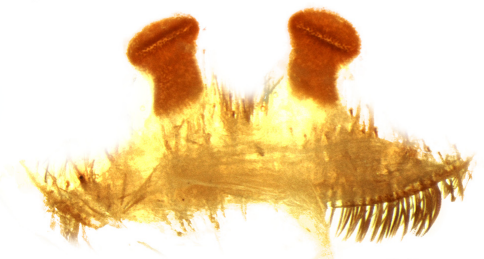*  *P. daweiensis* Sivayyapram et al., 2020 ♀ | *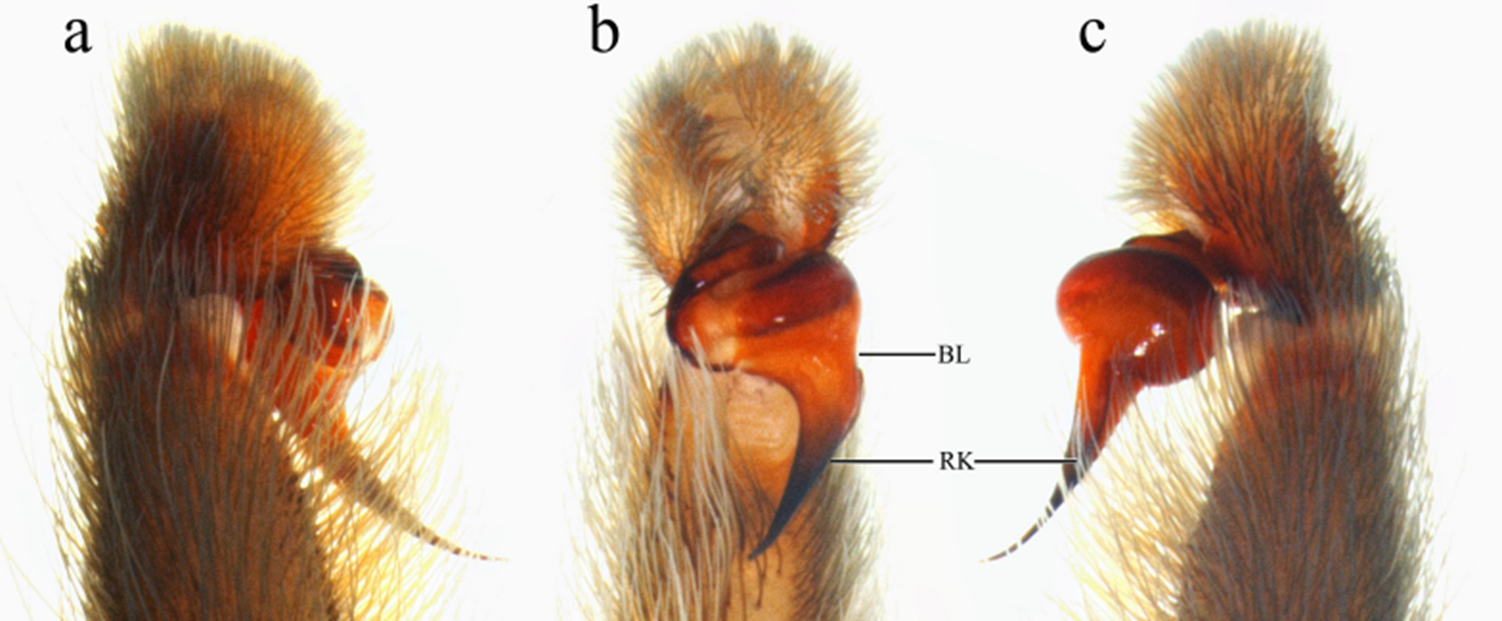*  *P. daweiensis* Sivayyapram et al., 2020 ♂ |
| 7 | 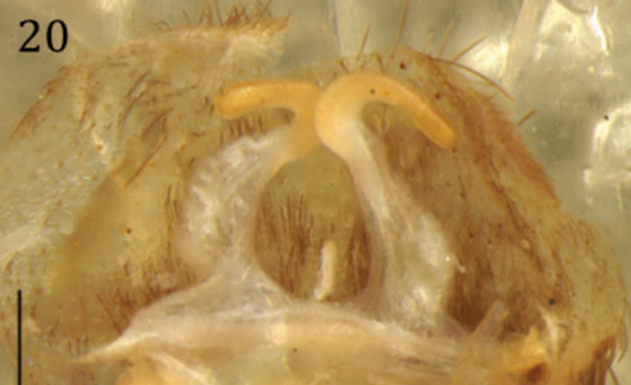  *P. longipalpus* Chomphuphuang et al., 2017 ♀ | *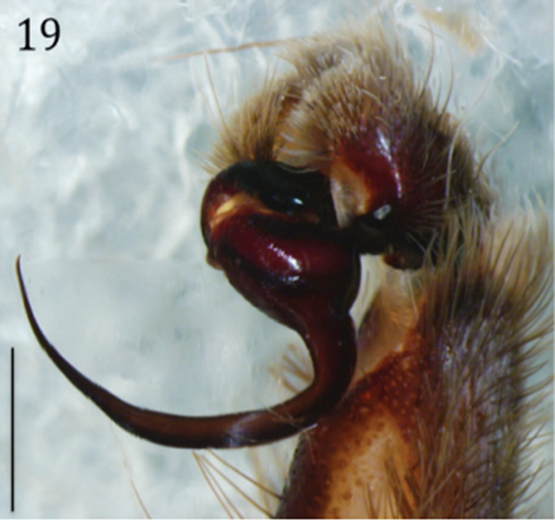*  *P. longipalpus* Chomphuphuang et al., 2017 ♂ |
| 8 | 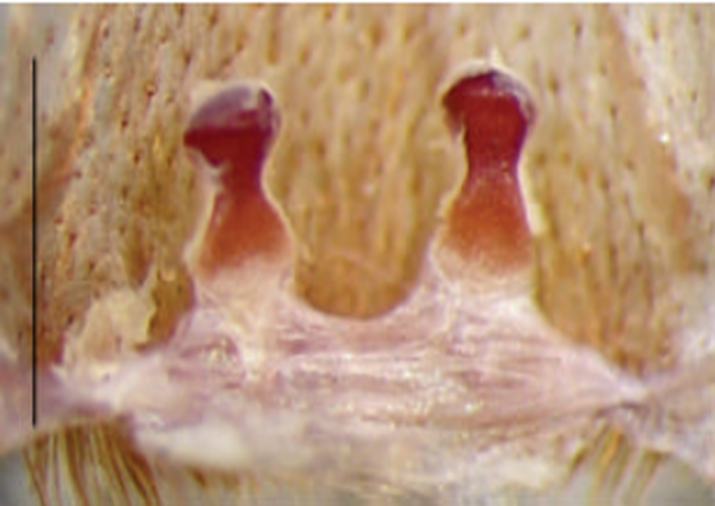  *P. moniqueverdezae* Nunn et al., 2016 ♀ | *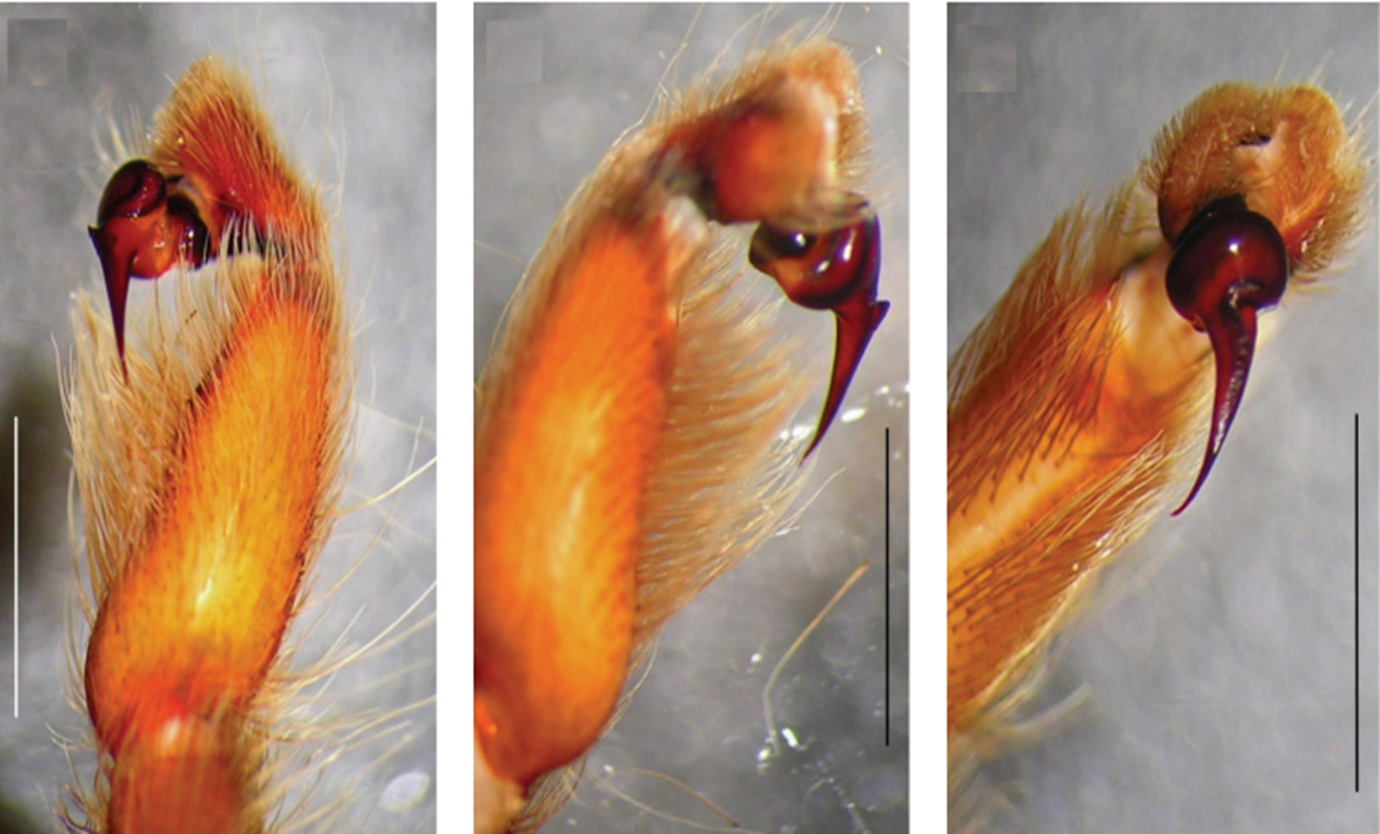*  *P. moniqueverdezae* Nunn et al., 2016 ♂ |
| 9 | *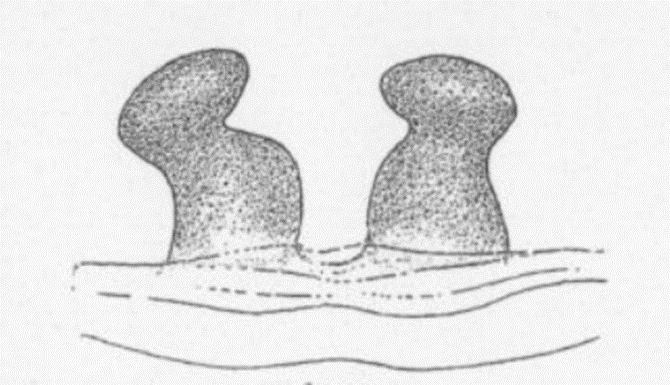*  *P. mutus* (Giltey, 1935) ♀ | N/A  *P. mutus* (Giltey, 1935) ♂ |
| 10 | *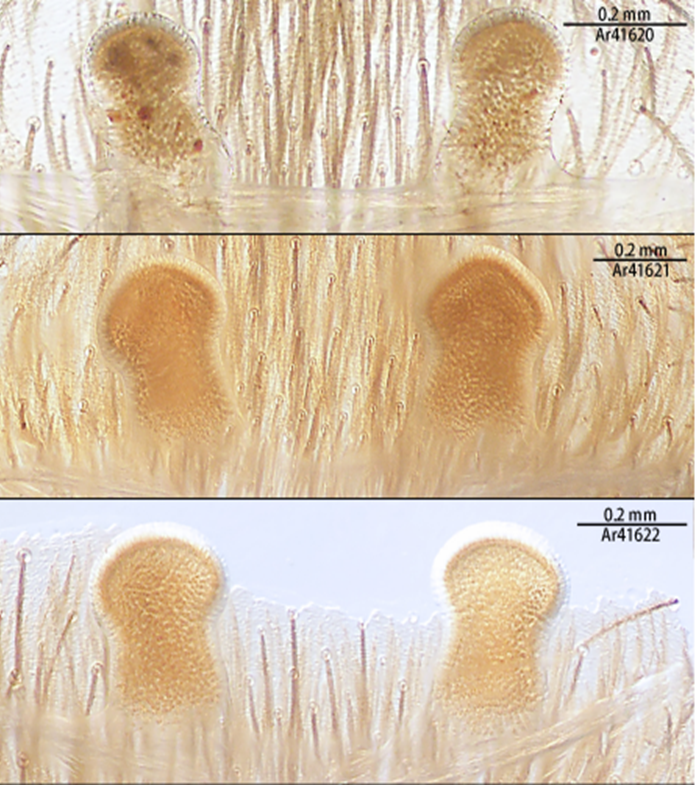*  *P. quanyui* Lin et al., 2021 ♀ | 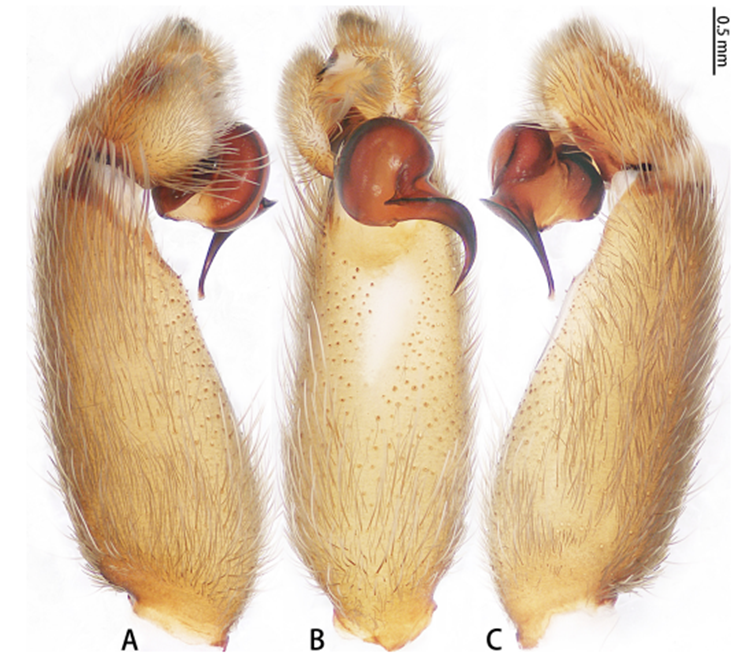*P. quanyui* Lin et al., 2021 ♂ |
| 11 | 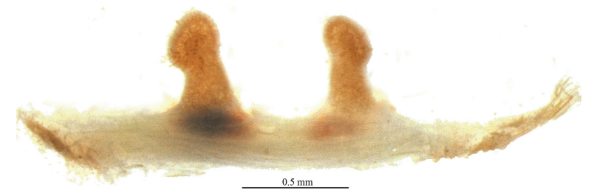  *P. raveni* Sivayyapram et al., 2020 ♀ | *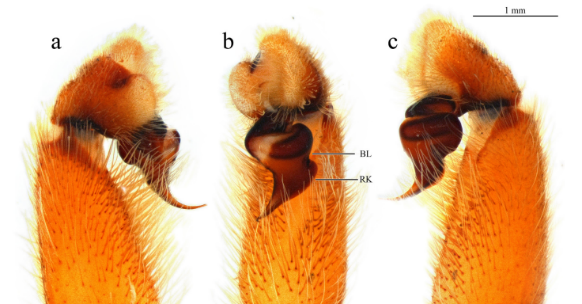*  *P. raveni* Sivayyapram et al., 2020 ♂ |
| 12 | *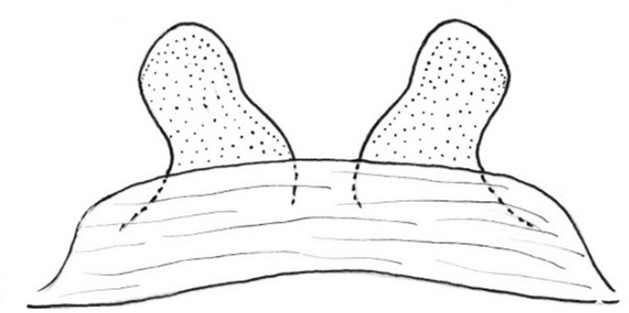*  *P. watasei* (Kishida, 1920) ♀ | 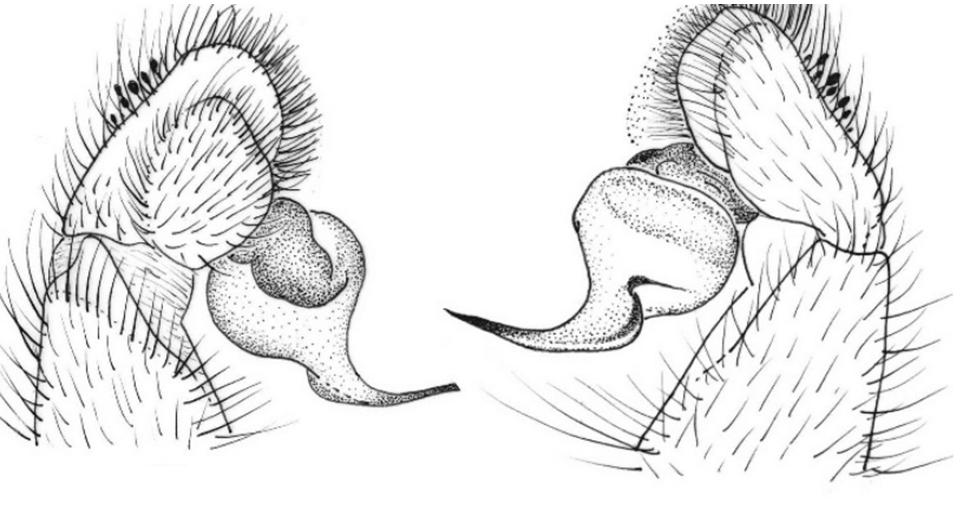  *P. watasei* (Kishida, 1920) ♂ |

**Table 2** GenBank accession number of *Phlogiellus khampheng* **sp. nov.** and *Phlogiellus* species in Thailand.

| No. | Species | Isolate | accession number |
| --- | --- | --- | --- |
| 1 | *Phlogiellus khampheng* **sp. nov.** | Pkp1 | PV454485 |
| 2 | *Phlogiellus khampheng* **sp. nov.** | Pkp2 | PV454486 |
| 3 | *Phlogiellus khampheng* **sp. nov.** | Pkp4 | PV454487 |
| 4 | *Phlogiellus khampheng* **sp. nov.** | Pkp5 | PV454488 |
| 5 | *Phlogiellus khampheng* **sp. nov.** | Pkp6 | PV454489 |
| 6 | *Phlogiellus khampheng* **sp. nov.** | A6 | PV454490 |
| 7 | *Phlogiellus moniqueverdezae* | Pmq1 | PV454491 |
| 8 | *Phlogiellus moniqueverdezae* | Pmq2 | In process submission |
| 9 | *Phlogiellus moniqueverdezae* | Pmq4 | PV454492 |
| 10 | *Phlogiellus moniqueverdezae* | Pmq5 | PV454493 |
| 11 | *Phlogiellus moniqueverdezae* | Pmq6 | In process submission |
| 12 | *Phlogiellus moniqueverdezae* | Pmq7 | PV454494 |
| 13 | *Phlogiellus moniqueverdezae* | Psp8 | PV454495 |
| 14 | *Phlogiellus moniqueverdezae* | Psp9 | PV454496 |
| 15 | *Phlogiellus longipalpus* | Plp2 | PV454497 |
| 16 | *Phlogiellus longipalpus* | Plp5 | In process submission |
| 17 | *Phlogiellus longipalpus* | Psp5 | PV454498 |
| 18 | *Phlogiellus longipalpus* | Psp6 | PV454499 |
| 19 | *Chilobrachys natanicharum* | Cnr1 | In process submission |

**Appendix**

Holotype, paratype and other material examined (museum numbers given where known):

**BMNH specimens:**

- 1 ♀ *Chilobrachys andersoni* Holotype, BMNH 1891.10.15.6, Mergui, A. Anderson, Musagetes andersonii
- 1 ♂ *Chilobrachys andersoni*, BMNH 1895.9.21.21, Southern Tenasserum, Burma, Phlogius cervinus
- 1 ♀, 5 J *Chilobrachys assamensis* Holotype, BMNH 1909.10.7.20-27, Assam, Peal
- 8 J *Chilobrachys assamensis*, BMNH 1909.10.7. 28-31, Assam, Peal
- 1 ♂ *Chilobrachys bicolor* Holotype, BMNH 1891.11.25.3, Kijouske, Burma, E.W. Oates
- 1 ♀ *Chilobrachys brevipes* Holotype, BMNH 1896.2.20.1, Tharrawaddy, Burma, E.W. Oates, Musagetes bevipes is synonymous with *Chilobrachys sericeus* det. A M Smith
- 1 ♂ *Chilobrachys femoralis* Holotype, BMNH 1899.11.2.23, Nasik, G.P. Millet
- 1 ♀ *Chilobrachys flavopilosus* Holotype, BMNH 1896.2.20.2, Tharrawaddy, Burma, E.W. Oates Musagetes decipiens
- 1 ♀ *Chilobrachys flavopilosus* Holotype, BMNH 1895.9.21.22, Thayetmyo, Upper Burma, E.W. Oates, Synonymous with *C. bicolor* Pocock 1895 Det. A M Smith 1990
- 1 ♀ *Chilobrachys hardwicki* Holotype, BMNH 1899.7.14.2, Chota Nagpur, Bengal, Rev. A. Logsdail
- 1 J *Chilobrachys oculatus* Holotype, BMNH 1895.9.21.41, Akyat, E.W. Oates
- 1 ♂ *Chilobrachys sericus* Holotype, BMNH 95.9.21.24, Rangoon, South Burma, E.W. Oates
- 1 ♂ 14J *Chilobrachys sericlus* Holotype, BMNH 1895.9.21.24 -39, Rangoon, South Burma, E.W. Oates
- 2 ♂ *Chilobrachys thorelli* Holotype, BMNH 1897.6.24.22-23, Sadiya, India, Godwin and Austen

**MNHN specimens:**

- AR4659 1 ♀ 5431 *Chilobrachys*, *Chilobrachys* sp, det. R. Gabriel 28/10/23
- AR4661 1 ♀ 14 imm *Chilobrachys nitelinus* Karsch, Kandy!, *Chilobrachys nitelinus* det. R. Gabriel 26/10/23
- AR4665 1 im ♂ 15 imm 25314 *Chilobrachys*, Kanandan, Sumatra, *Chilobrachys* sp det. R. Gabriel 26/10/23
- AR4666 1 juv *Chilobrachys tschankhoensis* Schenke, Type – det. 1946, Ichanh Hoa 10-III-25, imm of indeterminate sex det. D. Sherwood and R. Gabriel 05/12/19
- AR4667 1 ♀ 8 imm 35709 *Chilobrachys* Peradenyia 1914 (Buxton), *Chilobrachys* sp det. R. Gabriel 26/10/23
- AR4668 1 ♀ 21768 *Chilobrachys fumosus* Pocock, Bas pl. de l’Himalaya (Cartest), *Chilobrachys* sp det. R. Gabriel 26/10/23
- AR4696 1 ♂ 3278 *Chilobrachys*, Benkolen, *Chilobrachys* sp det. R. Gabriel 26/10/23
- AR4697 3 imm Selenocosminae, Dorey, Raffray, Selenocosminae det. R. Gabriel 26/10/23
- AR4698 1 ♀ 18633 *Chilobrachys*, Celebes S. Bua-Kraeng, 5000 (Febr, 1890), Fruhstorfer, *Chilobrachys* sp det. R. Gabriel 26/10/23
- AR4699 1 ♂ 1027 *Selenocosmia imbellis* E.S. (Phlogius) Borneo
- AR4701 15146 *Selenocosmia*, Java, Znider Mt (Tr. Gr.)
- AR4702 x 3 2284 *Selenocosmia*, M. Harmand, det. *Ornithoctonus* Raven, 04
- AR4703 1 ♀ 14416 *Selenocosmia effera* E, S., (Musee de Geneve), Java, 1903, *Selenocosmia effera*, g Boris Striffler 30/04/04
- AR4705 2 ♂ 4511 *Selenocosmia*, Java, (Ploem)
- AR4706 1 ♀ 18693 *Selenocosmia sumatrana* Thorell, Leimbrugge (Mengala), *Selenocosmia* sp det. R. Gabriel 14/11/24
- AR4700 2 ♀ 8 imm 22205 *Selenocosmia*, S. Java: Palabuan (Tr. Gr.), *Selenocosmia* sp det. R. Gabriel 20/10/23
- AR16707 1 ♀ *Selenocosmia ornata* E. Sim, Auct. Det, Siam Bocouit, *Melopoeus albostriatus* det. R. Gabriel 14/11/24
- AR16708 1 ♀ Saigon, A. Krempf, 1911, A. Krempf 1910 A.489, A. Krempf 489-A, *Chilobrachys* sp, det. R. Gabriel 14/11/24
- AR16712 1 imm ♀ *Phrictus* E, Simon zd., Sumatra, Luciaen Rochet 1880, *Selenocosmia* sp det. R. Gabriel 15/11/24
- AR16727 4 ♀ *Selenocosmia ornata* E. Sim Siam M’ Bocouit, 927/02, Auct. Det, *Melopoeus albostriatus* det. R. Gabriel 14/11/24
- AR17710 1 ♂ Presq. De Malacca Erington de la hoie et Chapie 1900, *Selenocosmia* sp det. R. Gabriel 14/11/24
- AR17786 1 ♀ 1 imm FE *Selenocosmia ornata* E. Sim, Cochinchina Germain, Auct. det., *Melopoeus albostriatus* det. R. Gabriel 14/11/24
- AR17793 1 ♀ *Selenocosmia harmandi* E. Simon. Auct. det (Julliet 1876), Ornithoctonidae, possible *M. albostiatum* no exact location, det. R. Gabriel 14/11/24
- AR17865 2 ♀ *Selenocosmia albostriata* E. Simon Auct. det. Cambodge Pavie 1885, *Melopoeus albostriatum*, det. R. Gabriel 07/24
- 2 ♀ 2 imm *Selenocosmia albostriata* E. Simon Auct. det, Siam Pavie 1885
- 1769 2♂ 2 imm. ♂ *Phlogiellus* sp Java: Buitenzong (Semin [sic] det. R. Gabriel 27/02/2025
- AR4671 1♀ *Phlogiellus baeri* E. S. Manila, det. R. Gabriel 27/02/2025
- AR 4672 1♀ *Phlogiellus* sp Borneo occ. Schmitao (Chaper) *Phlogiellus* sp det. R. Gabriel 27/02/2025
- AR4676 1♂ 1 imm. ♂*Phlogiellus inermis* Auss. Java (V. H.): TYPE, det. R. Gabriel 27/02/2025
- AR 4673 1♂ 1♀ *Phlogiellus inermis* Auss. Java! M. Gedi (Tr), det. R. Gabriel 27/02/2025
- AR 4674 1♀ 1 imm. ♂ *Phlogiellus* Aulipolo! det. R. Gabriel 27/02/2025
- AR4677 1♂ 2♀ 3 imm. *Phlogiellus* Java: Fissunapan 4000 (Tr), det. R. Gabriel 27/02/2025

**MSNG specimens:**

- 1♀ *Selenocosmia doriae* Thor: Sarawak, Borneo, Doria and Beccari
- 1♀ *Selenocosmia javanensis* Walk: Var B *sumatrana* Sumatra, Mount Singalang, VI. 1878 Beccari

**NHMW specimens:**

- 163 1 ♂ *Chilobrachys assamensis* Hirst, Indien: Assam, A.D. 15.X.1938, leg Gravely, det. Reimosu, 1ex. *Chilobrachys assamensis* *left palp dissected by previous worker missing, det. R. Gabriel & D. Sherwood 01/09/2023
- 164 1 ♀ *Chilobrachys formosus* Poc, Indien: Darjiling, A.D. 15.X.1938, leg Gravely, det. Reimosu, 1ex. *Chilobrachys* sp imm ♀? det. R. Gabriel & D. Sherwood 01/08/2023
- 165 1 ♀ *Chilobrachys sericius* (Thor), Birma: Rangoon, A.D. 15.X.1938, don. et. det. Reimosu, 1ex., NHMW 30306, *Chilobrachys* sp imm ♀ det. R. Gabriel & D. Sherwood 01/09/2023
- 71 2 ♀ 1 s/a ♂ *Selenocosmia javanensis* (W.), Java, Breitenstein 1882, det. R. Gabriel and A. Culmer 05/08/2022
- 173 1 ♀ *Selenocosmia javanensis*, Java, Novara. exp. det. Ausserer 1 ♀ 1866. 1 .47
- 173 1 ♂ *Selenocosmia javanensis*, Java, Novara. exp. det. Ausserer 1 ♀ 1866. 1 .47
- 173 5 2 s/a 3 juv *Selenocosmia javanensis*, Java, Novara. exp. det. Ausserer 1 ♀ 1866. 1 .47 [jar contains a 6th ex dried specimen Frfld 1862]
- 174 2 ♀ 1 ♂ *Selenocosmia javanensis*, Sumatra, leg. Hagen 3ex. *Selenocosmia* sp
- 175 *Selenocosmia javanensis*, Java, don, Moscovits, (Acqis. Nr. 1884. III.1,) Koelbel det: 4 ex (no 3 crossed out on label replaced by 4) 3 pinned 1 not pinned are these from same location? det. R. Gabriel and A. Culmer 05/08/2022
- 177 1 Imm ♂ 1 Imm ♀ *Selenocosmia javanensis* (W.), Amboina, 1858, leg. Doleschall, 2ex. Ambon (previously Amboina) – Maluku Islands- Indonesia det. R. Gabriel and A. Culmer 05/08/2022
- 178 1 ♂ *Selenocosmia javanensis* (W.), Borneo, Nanga Badan, leg, Bacles det. R. Gabriel and A. Culmer 05/08/2022
- 180 1 ♂ 1 s/a ♂ 1 s/a ♀ *Selenocosmia javanensis* (W.), Java: Britensarg, A.D. 15.X.1938, don. et. det. Reimosu, 1ex, det. R. Gabriel and A. Culmer 05/08/2022
- 181 *Selenocosmia javanensis* (W.), Sumatra, Padang, A.D. 15.X.1938, don. et. det. Reimosu, 4ex, det. R. Gabriel and A. Culmer 05/08/2022
- 182 Imm ♂ *Selenocosmia javanensis* (W.), Sumatra, Limaliu, A.D. 15.X.1938, don. et. det. Reimosu, leg. Jacobson, 1ex, previously dissected det. R. Gabriel and A. Culmer 05/08/2022
- 30210 1 ♀ 1 juv *Selenocosmia* sp, Avensamer, Java, det. Ray Gabriel 30/08/2023
- 30212 1 ♀ 1 juv ♂ Removed from mixed jar 30211 Werner Thielen leg., don. Lat Yao, 25km west Nakhon Sawann, Zeutral-Thailand, ca 80 uber 00, Acqu.-Nr.. 1989.XI.1., *Chilobrachys* sp, det. Ray Gabriel 30/08/2023, largest specimens in jar, Imm ♂ no hair, detached abdomen
- 30214 7 Imm *Phlogiellus* sp Padang, Sirambe, Sumatra (Grammostola like spermatheca) det. Ray Gabriel 30/08/2023
- 30217 2 ♀ *Selenocosmia* sp, Java: Britensarg, det. Ray Gabriel 30/08/2023
- 30228 1♂ *Phlogiellus* sp, det. Danni Sherwood 29/08/2023
- 30229 4 Imm Selenocosminae sp, det. Danni Sherwood 29/08/2023
- 30233 1♀ *Phlogiellus* sp, det. Danni Sherwood 29/08/2023
